# Targeted conversion of waste PET into dimethyl terephthalate and ethylene carbonate under metal-free conditions

**DOI:** 10.1016/j.eehl.2025.100139

**Published:** 2025-02-24

**Authors:** Minghao Zhang, Yijin Lu, Zhuo Wang, Xiong Gao, Xuanhang Luo, Xin Shen, Weixiang Wu, Qingqing Mei

**Affiliations:** a Institute of Environment Science and Technology, College of Environmental and Resource Sciences, Zhejiang University, Hangzhou 310058, China; b State Key Laboratory of Soil Pollution Control and Safety, Zhejiang University, Hangzhou 310058, China; c Key Laboratory of Environment Remediation and Ecological Health, Ministry of Education, College of Environmental Resource Sciences, Zhejiang University, Hangzhou 310058, China; d College of Agricultural and Environmental Sciences, University of California, Davis, CA 95616, USA

**Keywords:** Waste plastics, PET methanolysis, Dimethyl terephthalate, Ethylene carbonate, Ionic liquids, Hydrogen bonds

## Abstract

Ionic liquid-catalyzed methanolysis emerges as an efficient technique for transforming PET into premium-grade dimethyl terephthalate (DMT). However, incomplete depolymerization remains a major obstacle to the further industrial application of IL-catalyzed PET methanolysis. The proposed method utilized dimethyl carbonate (DMC) as the solvent for the complete methanolysis of waste PET under mild conditions, resulting in pure DMT and ethylene carbonate (EC) within 2.5 ​h. The use of 1-ethyl-3-methylimidazolium acetate ([EMIm][OAc]) as the IL catalyst significantly enhanced the reaction efficiency. Spectroscopic analyses using ^1^H NMR and FT-IR confirmed the pivotal role of [EMIm][OAc] in establishing multiple hydrogen bonds with the reactants (PET, DMC, and MeOH) and the intermediate [ethylene glycol (EG)] during the catalytic process. This catalytic system exhibited remarkable performance, achieving complete conversion of PET, which resulted in the production of DMT and EC with yields of 99% and 91%, respectively. Moreover, this versatile approach is applicable to the upcycling of a wide variety of commercial polyesters and polycarbonates, underscoring its potential as a comprehensive solution for plastic waste management.

## Highlights

• A comprehensive strategy for converting waste PET into dimethyl terephthalate and cyclic ethylene carbonate is proposed.
• This strategy features minimal methanol dosage and wide substrate scope harvesting various waste polyesters and polycarbonates.
• This upcycling method is highly efficient and selective, and the products can be easily separated and purified.
• Multiple H-bond intermediates during the reaction process plays a pivotal role in the catalytic process.
• The [EMIm][OAc] catalyst is efficient and environmentally friendly.

## Graphical abstract

**Figure:**
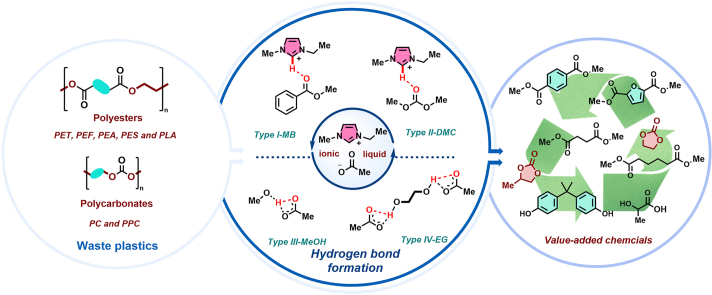

## 1 Introduction

The persistent accumulation of plastic waste, due to its inherent limited degradability and recyclability, remains a major cause of environmental damage and a critical threat to sustainable development [[1], [2], [3], [4]]. Polyethylene terephthalate (PET), the most consumed synthetic polyester plastic, has been one of the major plastic wastes [[5], [6], [7], [8], [9]]. The primary commercialized method for recycling PET is based on mechanical recycling. However, the suboptimal mechanical performance significantly restricts its versatility and effectiveness, leading to a gradual decline in product quality with each cycle [[9], [10], [11], [12]]. For instance, merely 7% of recycled PET is viable for repurposing into new bottles [10,13]. This issue highlights the pressing need for the development of effective strategies for handling plastics to ensure high-quality recycling.

**Fig. 1:**
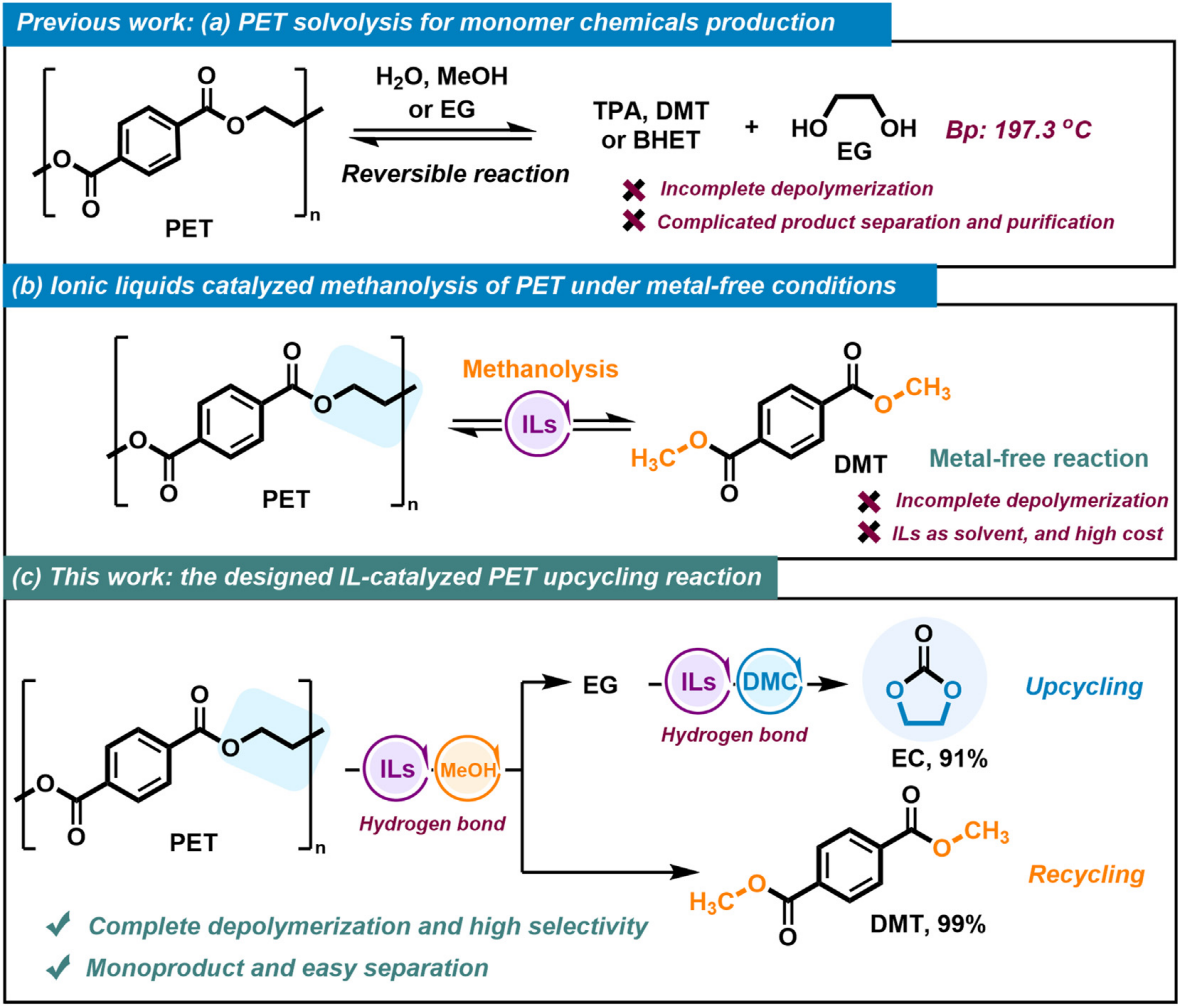
IL-catalyzed waste PET upcycling strategy in the methanolysis process.

Consequently, adopting and implementing an effective catalytic strategy for upgrading/recycling PET waste into its pristine starting monomers, such as terephthalic acid (TPA) or dimethyl terephthalate (DMT), holds significant potential for commercial operation [[14], [15], [16], [17], [18], [19], [20], [21], [22]]. These monomers can serve as raw feedstock to produce new high-quality commercial PET and biodegradable PBAT (polybutylene adipate terephthalate), demonstrating substantial potential economic benefits [[23], [24], [25]]. Currently, methanolysis is one of the most extensively studied methods for its great potential in industry application, and some of them have been verified on a pilot or commercial scale (Fig. 1a) [7,[26], [27], [28], [29]]. However, the presence of metal additives and undesired carboxylate by-products poses challenges to the progress of methanolysis, potentially increasing energy consumption in subsequent separation and purification processes [16,30].

Metal-free catalysis offers a promising solution for plastic recycling, bypassing concerns over metal catalyst residues and contamination [30,31]. Ionic liquids (ILs) emerge as a favorable catalyst option due to their inherent properties and catalytic efficacy in diverse chemical processes [[31], [32], [33], [34], [35]]. Recently, ILs have demonstrated outstanding catalytic efficacy in activating carbonyl and alcohol hydroxyl groups during PET alcoholysis [34,35], facilitated by synergistic interactions of the cations and anions. For example, 1-butyl-3-methylimidazolium acetate ([BMIm][OAc]) has been demonstrated to efficiently catalyze the methanolysis of polycarbonate (PC) [[36], [37], [38]], polyethylene 2,5-furandicarboxylate (PEF) [35] and PET [35]. However, most IL-catalyzed polyester methanolysis still encounter several challenges (Fig. 1b). Excessive ionic liquid usage, complexities in product isolation and purification, and especially incomplete depolymerization remain pressing concerns in polyester methanolysis (Table S1) [35,39]. Therefore, it is imperative to develop more efficient IL-based systems for achieving complete polyester methanolysis with lower catalyst dosages.

Herein, we reported a metal-free catalytic PET upcycling strategy, utilizing ILs as catalysts for the one-pot conversion of waste PET to DMT and ethylene carbonate (EC) (Fig. 1c). In this method, 1-ethyl-3-methylimidazolium acetate ([EMIm][OAc]) exhibited remarkable catalytic performance in the methanolysis of PET without any additives. This method successfully achieved complete conversion of PET, producing 99% DMT and 91% EC, respectively. The cations and anions of [EMIm][OAc] synergistically promote the methanolysis of PET and transesterification of ethylene glycol (EG) with dimethyl carbonate (DMC) via the formation of multiple H-bonds between reactants (MeOH, EG, PET, and DMC) and [EMIm][OAc]. Furthermore, various PET-based waste materials and other types of polyester and polycarbonate could also be upcycled, providing a practical approach to waste plastic recycling.

## 2 Materials and methods

### 2.1 Chemicals

Analytical grade solvents and commercially available reagents were purchased from commercial sources and used directly without further purification unless otherwise stated. PET powder (0.075 ​mm, made from Bottle-grade PET Chip, CR Chem-MAT CR-8863), poly(ethylene glycol succinate) (98%, Bide Pharmatech Ltd.), poly(ethylene adipate) (Mw: ∼1000, Aladdin), poly(ethylene 2,5-furandicarboxylate) (Alfa), polylactic acid (Mw: ∼60,000, Macklin), polycarbonate (Mw: ∼26,000, Macklin) and poly(propylene carbonate) (Mn: ∼5000, Rhawn), ethylene glycol (AR, Sinopharm), dichloromethane (AR, Shanghai Linfeng Chemical Reagent Co. Ltd.), MeOH (>99%, J&K Scientific), dimethyl succinate (99%, Meryer), dimethyl carbonate (>99%, Sinopharm), ethylene carbonate (99%, Meryer), furan-2,5-dicarboxylate (98%, Aladdin), dimethyl adipate (>99%, Bidepharm), [EMIm][Cl] (97%, Aladdin), [EMIm][OAc] (98%, Meryer), [EMIm][Br] (98%, Meryer), [EMIm][I] (97%, Rhawn), [EMIm][MeSO_3_] (98%, Meryer), [EMIm][BF_4_] (98%, Meryer), [BMIm][OAc] (98%, Meryer), CDCl_3_ (99%, Sigma–Aldrich), DMSO-*d*_6_ (99%, Adamas), and deionized water were used herein.

### 2.2 Experimental procedures

PET powder (1 ​mmol), [EMIm][OAc] (5% mol), MeOH (0.25 ​g, the weight ratio of MeOH to PET is 1.3), and DMC (3.5 ​mL) were added to a polytetrafluoroethylene rotor (25 ​mL), which was hermetically sealed in a stainless-steel reactor. The reactor was then stirred at 130 ​°C for 2.5 ​h on the heating plate with 500 r/min rotational speed. After completion of the reaction, the reaction system was quenched by moving the reactor into an ice bath. Then, the obtained reaction solution was diluted with 10 ​mL of dichloromethane. The liquid products were analyzed by gas chromatography (GC) using dodecane (0.5 ​mmol) as an internal standard. The yield of EC and DMT were calculated using the following equation:

$$\text{EC}\;\text{Yield}\;\left(\%\right)=\frac{\text{EC}\;\text{amount}\;\text{quantified}\;\text{by}\;\text{GC}\;\left(\text{mol}\right)}{\text{Theoretically}\;\text{Produced}\;\text{EC}\;\text{amount}\;\left(\text{mol}\right)}\times 100\%$$

$$\text{DMT}\;\text{Yield}\;\left(\%\right)=\frac{\text{DMT}\;\text{amount}\;\text{quantified}\;\text{by}\;\text{GC}\;\left(\text{mol}\right)}{\text{Theoretically}\;\text{Produced}\;\text{DMT}\;\text{amount}\;\left(\text{mol}\right)}\times 100\%$$

The reaction solution was first subjected to rotary evaporation to separate the volatile components, including the product (EC) and raw materials (DMC and MeOH), from the reaction mixture. Both the crude EC and the recovered crude DMC were subsequently isolated from the mixture through distillation. Meanwhile, highly pure and transparent DMT crystals were obtained through recrystallization and further washing with cold methanol. The product purity was characterized using NMR analysis (Figs. S13 and S16). The depolymerization of other polyesters and polycarbonates, including poly(ethylene succinate) (PES), poly(ethylene adipate) (PEA), PEF, polylactic acid (PLA), PC and poly(propylene carbonate) (PPC) was carried out in the same manner as the depolymerization of PET.

### 2.3 Materials characterizations

^1^H NMR and ^13^C NMR spectra were recorded at room temperature using a nuclear magnetic resonance (NMR) spectrometer (Bruker Avance-600, Bruker AG, Switzerland) (^1^H NMR at 600 ​MHz and ^13^C NMR at 151 ​MHz). NMR spectra of all products were reported in ppm with reference to solvent signals [^1^H NMR: CD(H)Cl_3_ (7.26 ​ppm), ^13^C NMR: CD(H)Cl_3_ (77.00 ​ppm)]. Signal patterns are indicated as s, singlet; d, doublet; dd, doublets of doublet; t, triplet, and m, multiplet. The detailed NMR characterizations and spectra of [EMIm][OAc], product DMT, and mixtures of [EMIm][OAc] with reactants (methyl benzoate, DMC, MeOH and EC) are provided in Figs. S4–S12. The product yield was analyzed by GC (Agilent 8860 and Agilent 7890A/5975C, Agilent, USA) (Agilent 8860 with Agilent J&W HP-5 Polysiloxane GC Column) and GC–mass spectrometry (GC–MS) (Agilent 7890A/5975C GC/MS with Agilent J&W HP-5 Polysiloxane GC Column). The DRIFT spectra were recorded at a spectral resolution of 2 ​cm^−1^ using a Fourier-transform infrared spectrometer FT-IR (Nicolet iS20, Thermo Fisher Scientific, USA). The FT-IR spectra of [EMIm][OAc] with MeOH or EG at different ratios were recorded using a quartz IR cell sealed with CaF_2_ windows connected with a vacuum system.

## 3 Results and discussion

### 3.1 Catalytic system exploration

**Fig. 2:**
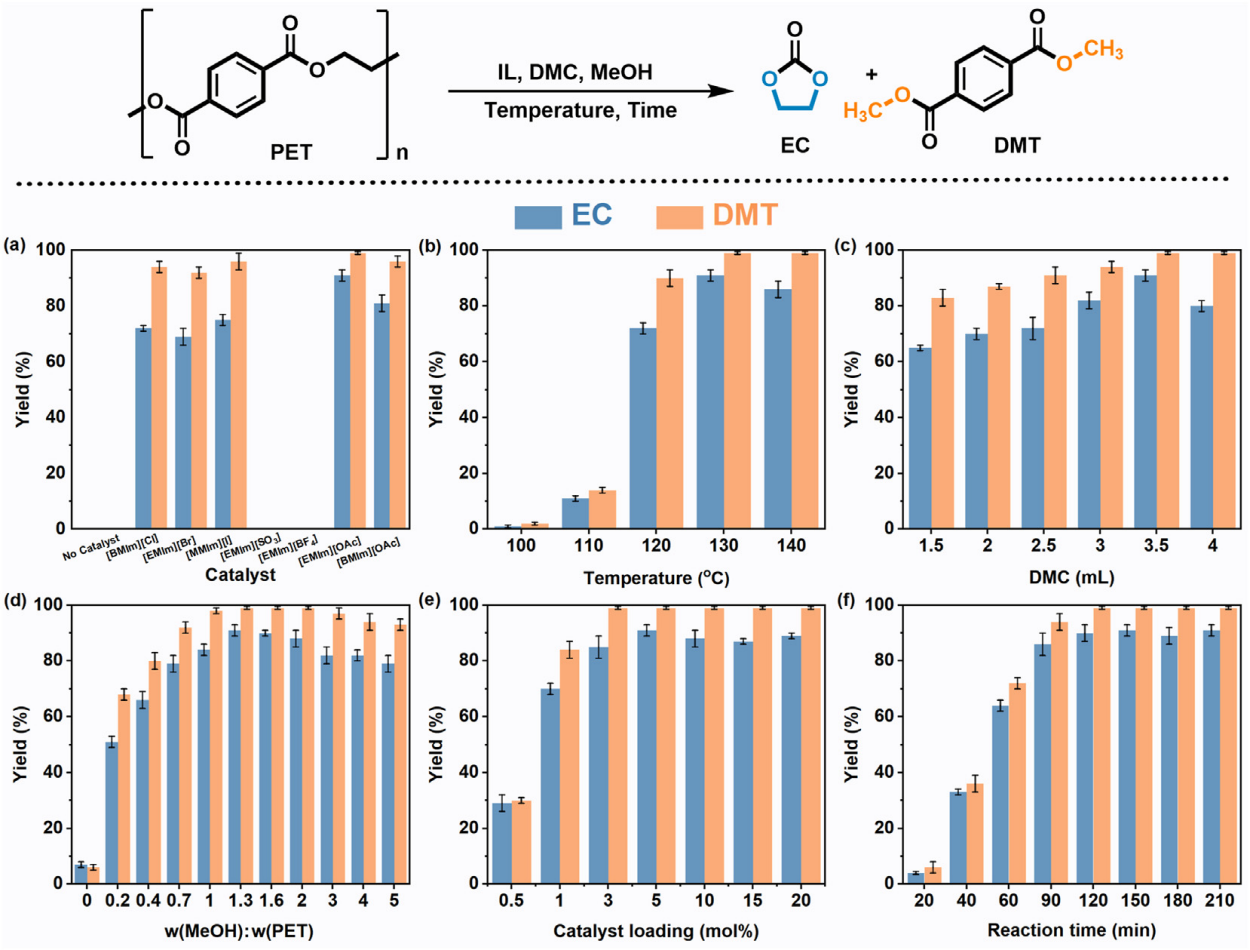
Catalytic system and reaction condition exploration. Standard reaction conditions: PET (1 ​mmol), MeOH [w(CH_3_OH):w(PET) ​= ​1.3:1], [EMIm][OAc] (5 ​mol%) and DMC (3.5 ​mL) at 130 ​°C for 2.5 ​h. Yields were determined by GC analyses with dodecane as an internal standard.

The sustained production of cyclic EC through PET upcycling involves PET methanolysis followed by the transesterification of EG with DMC. Our experimental approach involved using equivalent levels of MeOH as nucleophilic reagents and DMC as a trapping reagent for EG over a 2.5-h period at 130 ​°C. Surprisingly, in the absence of ILs, nearly all PET remained undegraded, underscoring the indispensable role of ILs in the depolymerization reaction. Prior studies have confirmed that the screening of anion H-bond acceptors directly influences the catalytic activity of ILs in the formation of relative hydrogen bonds [40]. Generally, the relative H-bond acceptance of anions follows the order of [OAc]^−^ ​> ​[MeSO_3_]^−^ ​> ​[X]^−^ ​> ​[BF_4_]^−^ [34,40,41]. To elucidate the impact of anions on the depolymerization process, 1-ethyl-3-methylimidazolium cation ([EMIm]^+^)-based ILs with various anions, including halogen anion (X^−^), [MeSO_3_]^−^, [BF_4_]^−^, and [OAc]^−^ were explored (Fig. 2a and Fig. S3). As anticipated, the data revealed that the [OAc]^−^, with stronger H-bond acceptance capacity, exhibited excellent catalytic properties, yielding 99% DMT and 91% EC (GC yield), respectively. Substituting anions led to a decreased yield of EC, or even the failure of PET depolymerization, highlighting the crucial role of the acetate anion in this PET upcycling reaction. The decrease in product yield primarily results from the diminished binding capacity of the anion with the hydroxyl hydrogen in alcohols (MeOH and EG), hindering the formation of H-bonds. This reduction in binding capacity subsequently lowers the nucleophilicity of the hydroxyl oxygens, resulting in inadequate PET depolymerization and limited EG cyclization with DMC. Furthermore, the catalytic activity of the 3-butyl-1-methylimidazolium cation ([BMIm]^+^) was found to be inferior to that of [EMIm]^+^, with the yield of EC decreasing to 81% (Fig. 2a). This disparity is probably due to a site-blocking effect that interferes with the formation of hydrogen bonds [41].

Subsequent variations in elevating or lowering the reaction temperature (based on 130 ​°C) showed a continuous decrease in EC yield (Fig. 2b). Temperature variation resulted in minimal changes in the selectivity of DMT; however, an increase in temperature caused a slight decrease in the selectivity of EC. Simultaneously, inadequate depolymerization of PET occurs at temperatures below 130 ​°C. The methanol-to-DMC ratio has a significant effect on the reaction. It was observed that reducing the amount of DMC resulted in a subsequent decrease in the yields of both DMT and EC (Fig. 2c). In addition, in the absence of MeOH, the yields of DMT and EC were only 7% and 6%, respectively, indicating that using only DMC as the reaction solvent and methylation reagent would lead to poor PET depolymerization (Fig. 2d). The addition of MeOH significantly enhances the yields of DMT and EC. This suggests that the presence of methanol introduces more nucleophilic reagents, which are readily activated by [EMIm][OAc] to attack the carbonyl group of PET, thereby facilitating the production of more monomers. The yields of DMT (99%) and EC (91%) were the highest when the added weight of MeOH to PET reached 1.3, where the complete methanolysis of PET and the efficient transesterification of EG with DMC were achieved. However, as the added weight of MeOH to PET was further increased to 5, the yields of both DMT and EC gradually decreased. These results imply that the influence of excess MeOH on PET methanolysis is minimal, but it can impede the EG transesterification to produce EC. Moreover, the impact of catalyst loading was also investigated, as depicted in Fig. 2e. Slightly diminished EG cyclization reactions with DMC were noted when inadequate or excessive amounts of [EMIm][OAc] were employed. Remarkably, using a mere 1 ​mol% loading of [EMIm][OAc], efficient PET depolymerization was also achieved, resulting in yields of 84% for DMT and 70% for EC, respectively.

To understand the progression of depolymerization, the time course of the reaction was also examined (Fig. 2f). It was observed that the yields of DMT and EC continuously increased with the increasing of reaction time, reaching their peak levels of 99% and 91%, respectively, after 2.5 ​h (Figs. S14 and S15). Compared with reported results using ILs or eutectic solvents as catalysts (Table S1), this approach exhibits remarkable catalytic activity for PET methanolysis at relatively lower reaction temperatures. Moreover, compared with typical PET methanolysis systems (Table S1), this work significantly reduces MeOH usage while achieving higher PET to DMT conversion rates, potentially lowering energy consumption in subsequent separations. Therefore, the devised IL-catalyzed PET methanolysis method harvests high yields of value-added product (DMT and EC) and consumes less volatile MeOH, which is expected to be a valuable solution for waste PET disposal.

### 3.2 Scope of substrates

**Fig. 3:**
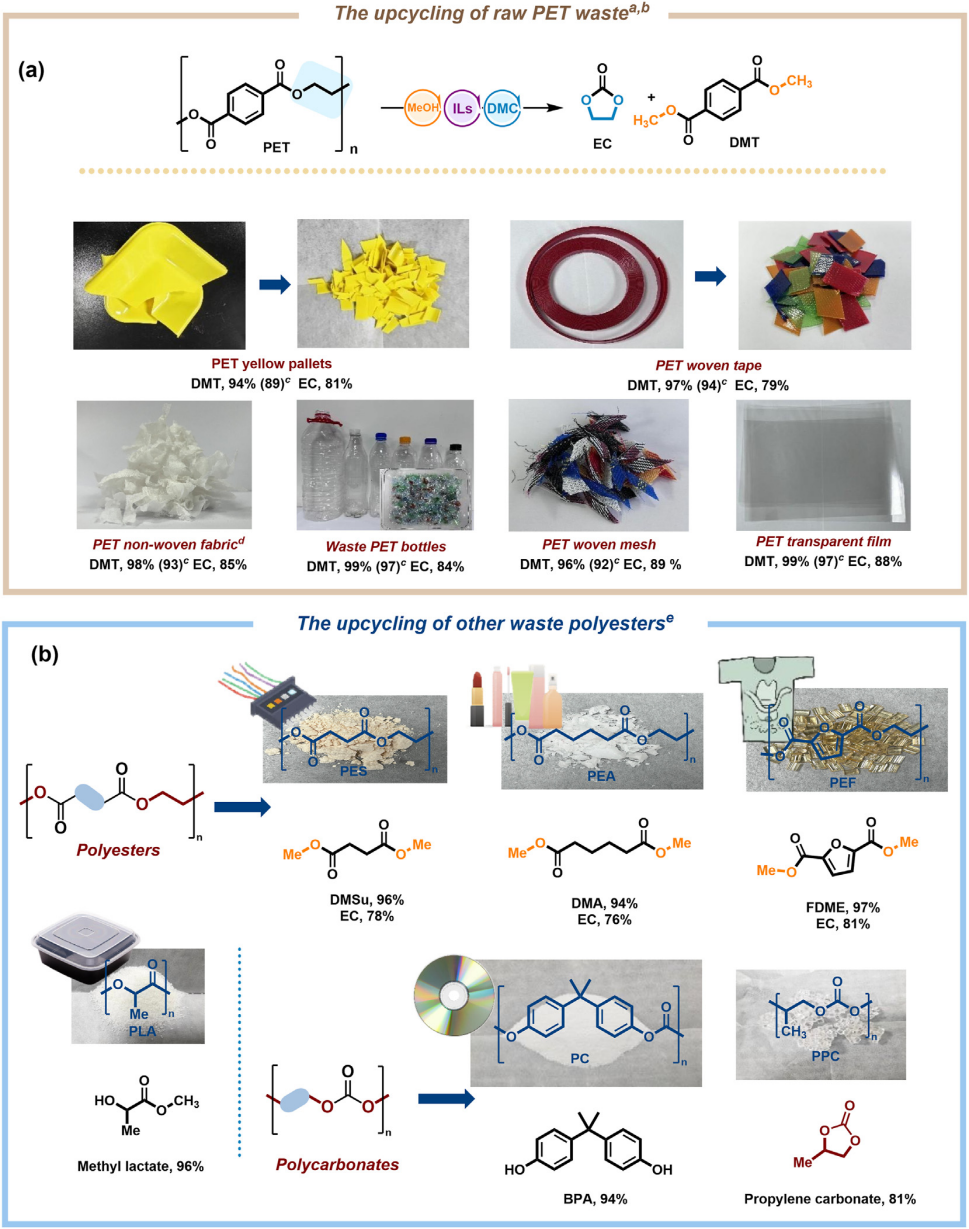
Scope of substrates. ^*a*^ Standard reaction conditions: polyester (1 ​mmol), MeOH [w(CH_3_OH):w(polyester) ​= ​1.3:1], [EMIm][OAc] (5 ​mol%), and DMC (3.5 ​mL) at 130 ​°C for 3 ​h ^*b*^ Yields were determined by GC analyses with dodecane as the internal standard. ^*c*^ Isolated yields. ^*d*^ At 130 ​°C for 4 ​h ^*e*^ Yields were determined by ^1^H NMR analyses with mesitylene as internal standard.

With the establishment of optimal reaction conditions, the scope of this study was expanded to evaluate the feasibility of converting a variety of commercially discarded or recycled PET wastes, such as pallets, non-woven fabric, transparent film, woven mesh, PET bottles, and woven tape. This assessment confirmed yields of 90%–99% for DMT and 79%–89% for EC. Fig. S1 illustrates the comparison of the reaction mixture before and after the reaction, as well as the isolated yields of DMT. Furthermore, the applicability of our innovative approach to various commercially available polyester and polycarbonate wastes was evaluated, including PES, PEA, PEF, PLA, PC, and PPC. As depicted in Fig. 3, our solution successfully converted these polyesters and polycarbonate wastes into dicarboxylates and five-membered cyclic carbonates, yielding dimethyl succinate (DMSu), dimethyl adipate (DMA), dimethyl furan-2,5-dicarboxylate (FDME), methyl lactate, and bisphenol A (BPA) in impressive 94%–97% yields, respectively. Meanwhile, EC or propylene carbonate was harvested with a yield of 76%–81%, respectively.

### 3.3 Controlled experiments and reaction kinetics

**Fig. 4:**
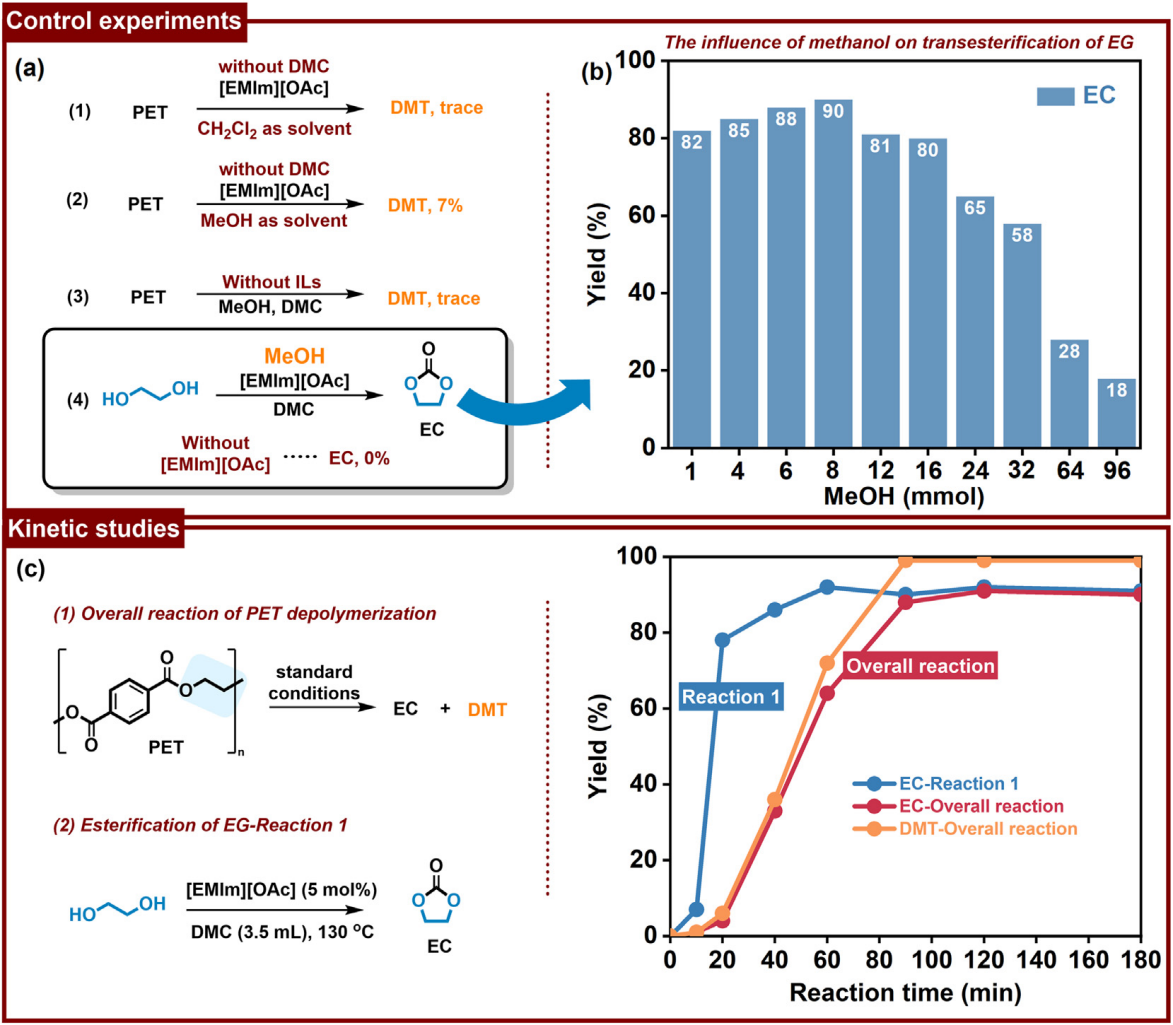
Control experiments and kinetic studies. (a) Control experiments. (b) The influence of MeOH on transesterification of EG. Reaction condition: EG (1 ​mmol), [EMIm][OAc] (5 ​mol%), and DMC (3.5 ​mL) at 130 ​°C. Yield detected by GC with dodecane as internal standard. (c) Kinetic studies. Standard reaction condition of 1: PET (1 ​mmol), [EMIm][OAc] (5 ​mol%), w(CH_3_OH):w(PET) ​= ​1.3:1, and DMC (3.5 ​mL) at 130 ​°C. Condition of reaction 2: EG (1 ​mmol), [EMIm][OAc] (5 ​mol%), and DMC (3.5 ​mL) at 130 ​°C.

To unravel the intricate relationships among experimental variables, a series of controlled experiments was conducted (Fig. 4 and Fig. S2). Using dichloromethane or methanol as solvents, PET methanolysis was conducted at 130 ​°C over a duration of 2.5 ​h without DMC. Negligible depolymerization of PET was observed, evidenced by the mere trace amount of DMT identified in the reactions (Fig. 4a, entries 1 and 2). This finding indicates that it is critical to shift the equilibrium in favor of DMT production by promoting EG transesterification with DMC, as depicted in Fig. 4a (entry 3). The absence of [EMIm][OAc] resulted in almost a failure of PET depolymerization and EC production, underscoring the indispensable catalytic role of [EMIm][OAc] in the reaction sequence (Fig. 4a, entries 3 and 4). The transesterification of EG with DMC was conducted using various amounts of MeOH. As depicted in Fig. 4b, with [EMIm][OAc] as a catalyst, the yield of EC remained unaffected when the MeOH dosage was below 8 ​mmol. However, with an increase in MeOH dosage, a notable decrease in EC yield was observed. With 94 ​mmol MeOH, only 18% EC was detected at 130 ​°C for 2.5 ​h. The decrease in EC yield aligns with previous results (Fig. 2d), suggesting that excess MeOH suppresses the transesterification of DMC with EG.

In addition, reaction kinetics were studied in depth to investigate the reaction pathways. Fig. 4c illustrates the time course of both the overall reaction and the sole EG transesterification process. With the remarkable catalytic activity of [EMIm][OAc], the EG transesterification process exhibited exceptional efficiency, achieving a noteworthy 92% yield of EC at 130 ​°C within 1 ​h. The transesterification of EG with DMC outpaces both the overall reaction and sole PET methanolysis under identical conditions, suggesting PET methanolysis as the rate-determining step in this system. Simultaneously, the significantly higher overall reaction rate compared to sole methanolysis highlights the substantial acceleration of PET methanolysis through the transesterification of EG with DMC, ultimately leading to the complete conversion of PET into DMT. Overall, these results indicated that the transesterification of intermediate EG with DMC shifts the reaction equilibrium, contributing to the high yields of DMT and EC.

### 3.4 Key roles of hydrogen bonds

**Fig. 5:**
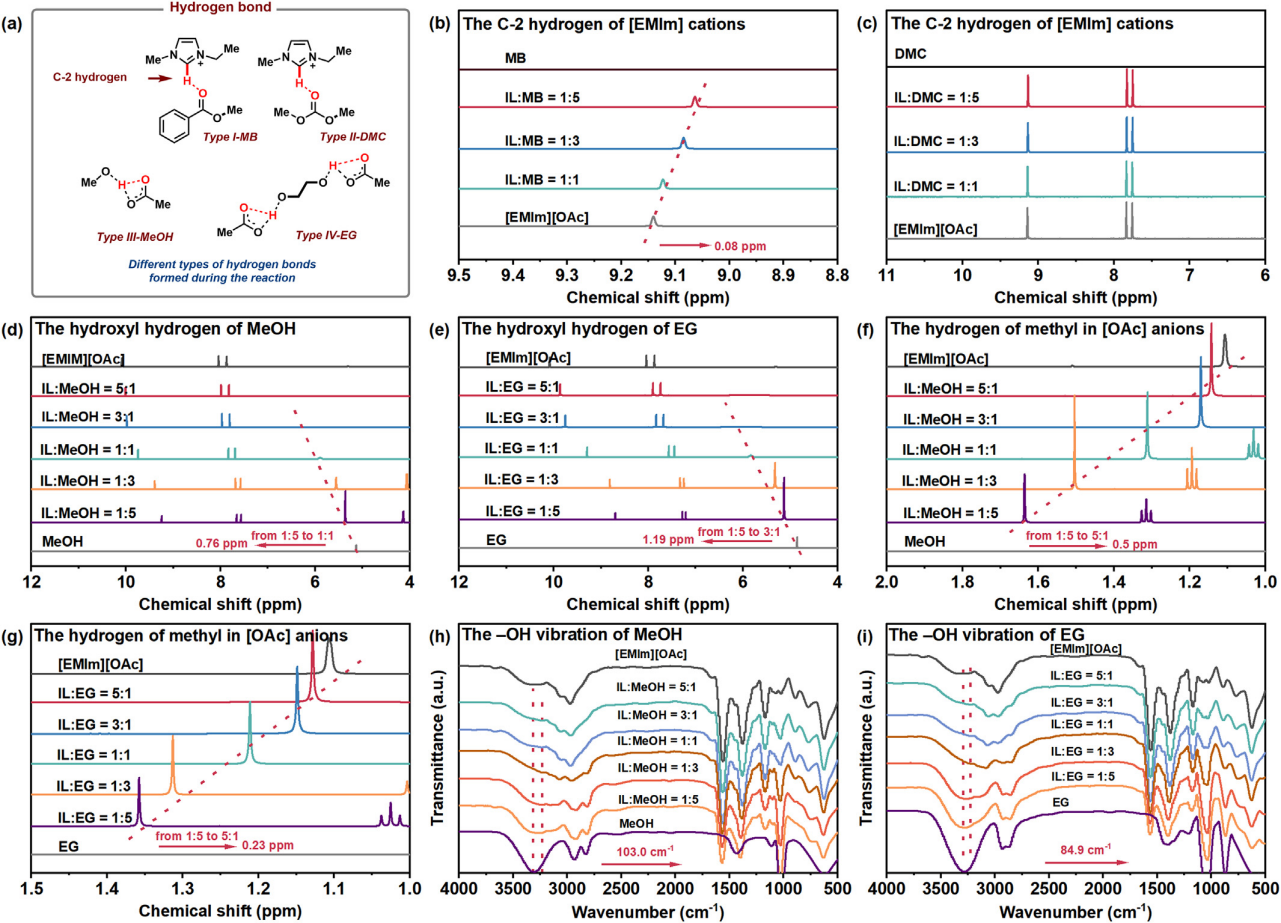
Characterization of different hydrogen bonds formed during the reaction. (a) Different types of hydrogen bonds formed during PET depolymerization. (b–g) ^1^H NMR spectra of the [EMIm][OAc] and different reactants mixture with different molar ratios. The ^1^H NMR spectra were recorded at room temperature in DMSO-*d*_6_. (h–i) FT-IR spectra of [EMIm][OAc] and alcohol mixtures of different molar ratios.

Based on previous relevant studies [34,35,[42], [43], [44]], the cleavage of PET is mainly induced by the hydrogen bonds between ILs and reactants. The proposed process may entail the formation of multiple hydrogen bonds between the [EMIm][OAc] and reactants during both PET methanolysis and EG transesterification (Fig. 5a, hydrogen bonds, Type I-IV). To verify this conjecture, MeOH, EG, DMC, and methyl benzoate (MB) were employed as probe molecules, and their interactions with [EMIm][OAc] were studied via ^1^H NMR analysis, as depicted in Fig. 5.

The interaction between [EMIm]^+^ and the carbonyl oxygen of reactants (MB and DMC) was first studied (Fig. 5b and c). In the ^1^H NMR spectrum of MB and [EMIm][OAc] mixture, the C-2 hydrogen of [EMIm]^+^ was consistently shifted towards high-field as the concentration of MB increased (Fig. 5a, hydrogen bond, Type I-MB) [35]. Specifically, the chemical shift of the C-2 hydrogen of [EMIm]^+^ moved from 9.14 to 9.06 ​ppm when the [EMIm][OAc] and MB ratio was 1:0 and 1:5, respectively. This shift indicated a robust hydrogen bond formation between the C-2 hydrogen of [EMIm]^+^ and the carbonyl oxygen of MB (Fig. 5a and b, Fig. S9) [35]. The activation of the carbonyl carbon in PET not only enhances its susceptibility to nucleophilic attack but also stabilizes the associated intermediates, thereby effectively triggering PET chain cleavage [35]. Surprisingly, in the spectrum of DMC and [EMIm][OAc], the C-2 hydrogen shift of [EMIm]^+^ was not obvious, which may be attributed to the insolubility of DMC and [EMIm][OAc] (Fig. 5c and Fig. S10).

Subsequently, the interactions between the hydroxyl hydrogen of alcohols (MeOH and EG) and [OAc]^-^ were investigated. The results suggested a gradual movement of the hydroxyl hydrogen signal for both MeOH and EG towards the downfield, accompanied by a broadening and eventual near disappearance of the peak with increasing concentration of [EMIm][OAc] (Fig. 5d and e) [34,35]. Specifically, in the mixed solutions of MeOH and [EMIm][OAc], the hydroxyl hydrogen signal peak underwent a shift from 5.13 to 5.89 ​ppm as the molar ratio of [EMIm][OAc] to MeOH increased from 0:1 to 1:1 (Fig. S11). Furthermore, this single peak nearly disappeared as the concentration of the [EMIm][OAc] continued to increase (Fig. 5d). The hydroxyl protons of EG moved from 4.85 to 5.84 ​ppm, and further to 6.04 ​ppm when the molar ratio of EG and [EMIm][OAc] was 1:0, 1:1, 1:3, respectively (Fig. 5e and Fig. S12). Meanwhile, the chemical shifts of hydrogen in [OAc]^−^ in both the mixed systems of [EMIm][OAc] and MeOH or EG further confirm the above suggestions [34,35,44]. Specifically, in the ^1^H spectrum of a mixture of [EMIm][OAc] and MeOH, the signal peak of the hydrogen of [OAc]^−^ shifted towards high-field from 1.64 ​ppm to 1.14 ​ppm when the molar ratio of [EMIm][OAc] to CH_3_OH increased from 1:5 to 5:1. Similar phenomena were observed in the IL and EG mixture, as depicted in Fig. 5g. These results indicated a robust hydrogen bond formation between the hydroxyl group of alcohols and the oxygen of acetate anions in [EMIm][OAc] (Fig. 5a, hydrogen bond, Type III-MeOH and Type IV-EG) [34,35,44].

The active interaction of MeOH or EG with [EMIm][OAc] is further demonstrated by FT-IR analysis. As the molar ratio of [EMIm][OAc] versus reactants (MeOH or EG) increased from 1:5 to 5:1, the –OH vibration of both MeOH and EG gradually broadened and nearly disappeared in the FT-IR spectrum (Fig. 5h and i). The redshift of –OH in MeOH reached 103.0 ​cm^−1^, from 3317.3 to 3214.3 ​cm^−1^ (Fig. 5h), while the redshift of –OH in EG was 84.9 ​cm^−1^, from 3291.1 to 3206.2 ​cm^−1^ (Fig. 5i). The observed redshifts were ascribed to the formation of robust hydrogen bonds between the oxygen atom of the acetate anion in [EMIm][OAc] and the hydrogen atom of the hydroxyl group in both MeOH and EG, consistent with reported results [34,35]. In summary, the extensive experimental studies support the hypothesis that [EMIm][OAc] activates MeOH and EG by enhancing the electronegativity of the hydroxyl oxygen, thereby enabling more favorable nucleophilic attacks to C=O of the ester group (PET and DMC) during the methanolysis of PET and transesterification of EG with DMC, respectively. The characterization of these hydrogen bonds between ILs and reactants further supported the hypothesis regarding their significance in both depolymerization of PET and enhancing the yields of both DMT and EC (Fig. 2) [35].

### 3.5 Reaction mechanism

**Fig. 6:**
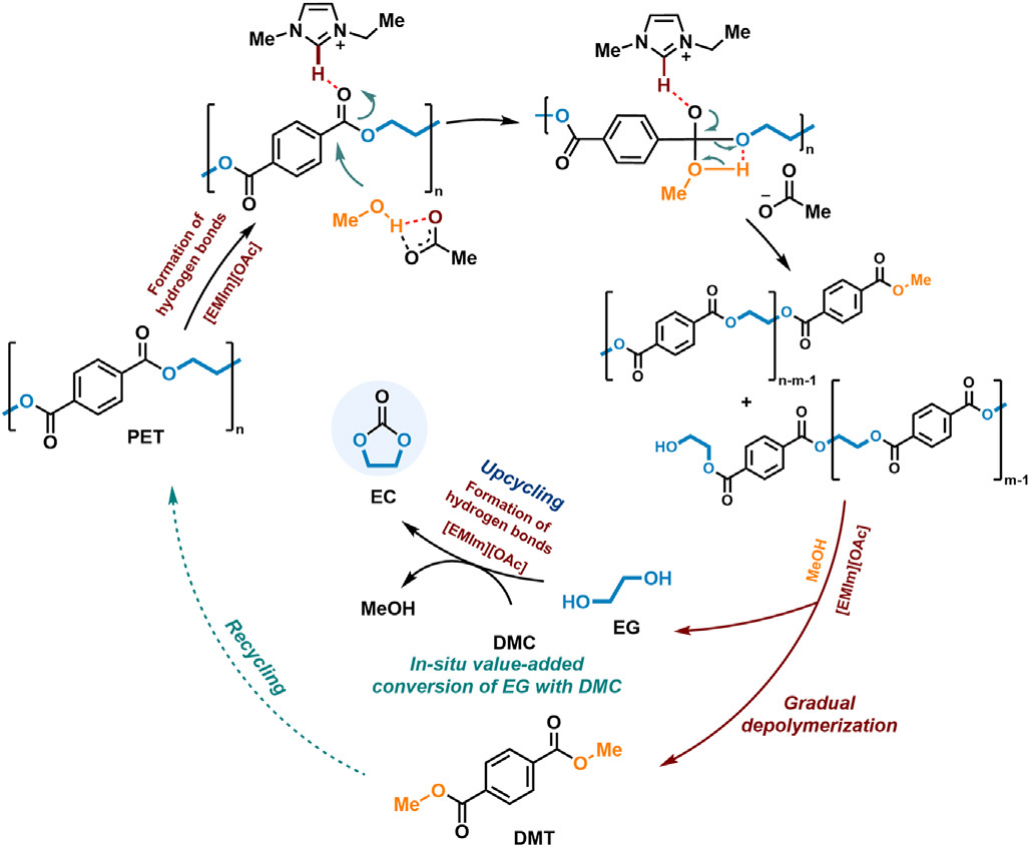
The possible reaction mechanism of PET methanolysis coupled with *in situ* EG upgradation into cyclic EC catalyzed by [EMIm][OAc].

Combining previously reported findings and our latest experimental results [26,34,35,44], we unveil a plausible chemical upcycling mechanism facilitated by [EMIm][OAc], as illustrated in Fig. 6. Initially, the anion and cation of [EMIm][OAc] serve a dual role, concurrently activating MeOH and the oxygen within the carboxyl group of PET through the formation of hydrogen bonds. Facilitated by these hydrogen bonds, activated MeOH, acting as a nucleophilic reagent, attacked the carbon of carbonyl in PET, forming a tetrahedral intermediate that gradually breaks down into smaller oligomers. The oligomer fragmentation process continuously produced DMT and EG monomers. Simultaneously, EG and DMC were activated in a similar manner through hydrogen bonding with the anion and cation of the IL, respectively. This process enhances the nucleophilic attack of EG with the carbonyl carbon in DMC to harvest EC. These dual promotions in both methanolysis and EG upgradation ensure a more efficient conversion process, underscoring the multifaceted role of [EMIm][OAc] in promoting the overall upcycling mechanism.

## 4 Conclusion

This study proposed a practical and facile approach for upcycling waste PET to assemble a variety of methyl dicarboxylate and EC. Benefitting from the exceptional catalytic efficiency of [EMIm][OAc], the reaction achieved complete conversion of PET within 2.5 ​h, yielding 99% DMT and 91% EC. NMR and IR analysis demonstrated that [EMIm][OAc] catalyzed the transesterification and cyclization reactions via hydrogen bonding, where the cation and anion activate the carboxyl groups (PET) and hydroxyl groups (MeOH and EG), respectively. Based on these results, a feasible catalytic mechanism for this PET upcycling route was proposed. Furthermore, this methodology has been effectively applied to the valorization of diverse waste PET materials and other polyesters (main products yield ≥ 94%, EC yield ≥ 76%), providing an accessible avenue for the transformation of waste PET. Future efforts should focus on the development of highly efficient and easily recyclable heterogeneous catalysts to simultaneously reduce costs and mitigate the environmental impact of industrial applications. Overall, this work holds significant potential for broad implementation within the polymer recycling industry.

## CRediT authorship contribution statement

**Minghao Zhang:** Writing – original draft, Investigation, Formal analysis. **Yijin Lu:** Formal analysis. **Zhuo Wang:** Writing – review & editing, Formal analysis. **Xiong Gao:** Formal analysis. **Xuanhang Luo:** Formal analysis. **Xin Shen:** Formal analysis. **Weixiang Wu:** Resources, Formal analysis. **Qingqing Mei:** Writing – review & editing, Supervision, Resources, Funding acquisition, Formal analysis, Conceptualization.

## Declaration of competing interests

The authors declare no competing interests.
