# Supplementary material for: Targeted conversion of waste PET into dimethyl terephthalate and ethylene carbonate under metal-free conditions
Source: Eco Environ Health. 2025 Feb 24;4(2):100139. doi: 10.1016/j.eehl.2025.100139 (PMC11985011; doi:10.1016/j.eehl.2025.100139)
Supplement: Multimedia component 1 [file mmc1.docx]

Supporting Information

Targeted conversion of waste PET into dimethyl terephthalate and ethylene carbonate under metal-free conditions

Minghao Zhang ^a^, Yijin Lu ^a^, Zhuo Wang ^a^, Xiong Gao ^a^, Xuanhang Luo ^a^, Xin Shen ^d^, Weixiang Wu ^a,b,c^, Qingqing Mei ^a,b,c,^*

*^a^*Institute of Environment Science and Technology, College of Environmental and Resource Sciences, Zhejiang University, Hangzhou 310058, China.

*^b^*Key Laboratory of Environment Remediation and Ecological Health, Ministry of Education, College of Environmental Resource Sciences, Zhejiang University, Hangzhou 310058, China.

*^c^*Zhejiang Province Key Laboratory for Water Pollution Control and Environmental Safety Technology, Hangzhou 310058, China.

*^d^*College of Agricultural and Environmental Sciences, University of California, Davis, California 95616, USA.

*Corresponding author.

E-mail address: [meiqq@zju.edu.cn](mailto:meiqq@zju.edu.cn) (Q. Mei)

**Table of Contents**

[1. Methanolysis of PET using different catalysts. 3](#_Toc7911)

[2. Converting commercial PET waste into DMT. 5](#_Toc4370)

[3. The effect of water content on the catalytic system of PET depolymerization. 6](#_Toc21581)

[4. Characterization of the hydrogen bonds formed during the processes. 7](#_Toc8279)

[4.1. The structures of ILs used in this study. 7](#_Toc4661)

[4.2.](#_Toc13593) ^[1](#_Toc13593)^[H NMR characterizations of reactants. 8](#_Toc13593)

[4.3.](#_Toc13222) ^[1](#_Toc13222)^[H NMR characterization of obtained DMT. 17](#_Toc13222)

[4.4.](#_Toc20544) ^[1](#_Toc20544)^[H NMR characterization of products. 18](#_Toc20544)

[5.](#_Toc2018) ^[1](#_Toc2018)^[H NMR and](#_Toc2018) ^[13](#_Toc2018)^[C NMR data of all compounds. 19](#_Toc2018)

[6.](#_Toc30233) ^[1](#_Toc30233)^[H NMR and](#_Toc30233) ^[13](#_Toc30233)^[C NMR spectra of all compounds. 20](#_Toc30233)

[References 21](#_Toc29642)

**1. Methanolysis of PET using different catalysts.**

Table S1. Methanolysis of PET with reported catalysts.

| Entry | Catalysts | MeOH:PET | Co-solvent | Temp. (℃) | T (h) | Conv. (%) | Yield (%) | Ref. |
| --- | --- | --- | --- | --- | --- | --- | --- | --- |
| 1 | AlP | n(MeOH:PET) = 76:1 | Toluene | 200 | 2 | - | 88.5 | [1] |
| 2 | Pb(AC)_2_+ Zn(AC)_2_ | w(MeOH:PET) = 2.8:1 | - | 120 | 2 | 97.8 | 97.8 | [2] |
| 3 | ZnO nanodispersions | w(MeOH:PET) = 6:1 | - | 170 | 0.25 | 97 | 95 | [3] |
| 4 | K_2_CO_3_ | n(MeOH:PET) = 50:1 | DCM | 25 | 24 | 100 | 93.1 | [4] |
| 5 | Orange peel ash | w(MeOH:PET) = 7.9:1 |  | 200 | 1 | - | 79 | [5] |
| 6 | LiOMe | w(MeOH:PET) = 3.9:1 | DMC | 65 | 5 | - | 91 | [6] |
| 7 | Bamboo leaf ash | n(MeOH:PET) = 49.4:1 | - | 200 | 2 | 100 | 78 | [7] |
| 8 | Ti_0.5_Si_0.5_O_2_ | w(MeOH:PET) = 7.8:1 | - | 160 | 2 | 100 | 98.2 | [8] |
| 9 | Calcined sodium silicate | w(MeOH:PET) = 5:1 | - | 200 | 0.5 | 100 | 95 | [9] |
| 10 | MgO/NaY | w(MeOH:PET) = 6:1 | - | 200 | 0.5 | 99 | 91 | [10] |
| 11 | ChCl/Zn(OAc)_2_ | w(MeOH:PET) = 2.5:1 | MeCN | 170 | 1 | 100 | 90.1 | [11] |
| 12 | [HDBU][Im] | w(MeOH:PET) = 0.8:1 | - | 140 | 3 | 100 | 75 | [12] |
| 13 | PIL-Zn^2+^ | w(MeOH:PET) = 4:1 | - | 170 | 1 | 100 | 90.3 | [13] |
| 14 | [HO_3_S-(CH_2_)_3_-NEt_3_]Cl[ZnCl_2_]_0.67_ | w(MeOH:PET) = 4:1 | - | 195 | 0.5 | - | 78.4 | [14] |
| 15 | [BMIm][OAc] | w(MeOH:PET) = 4:1 | - | 150 | 4 | 88.5 | 41.7 | [15] |
| 16 | [EMIm][OAc] | w(MeOH/PET) = 0.7:1 | - | 130 | 2.5 | 100 | 92 | This work |
| 17 | [EMIm][OAc] | w(MeOH/PET) = 1:1 | - | 130 | 2.5 | 100 | 98 | This work |
| 18 | [EMIm][OAc] | w(MeOH/PET) = 1.3:1 | - | 130 | 2.5 | 100 | 99 | This work |

As illustrated in Table S1, the catalytic performance of [EMIm][OAc] in PET methanolysis has been studied in detail and compared with catalysts previously disclosed in the literature. Based on these studies, many PET methanolysis processes frequently encounter challenges in effectively converting PET into DMT and low-value EG, such as harsh reaction conditions and incomplete depolymerization, especially low value of EG. Among these catalytic strategies, ionic liquids, facilitated by synergistic interactions between cations and anions, have demonstrated exceptional catalytic efficacy in activating carbonyl and alcohol hydroxyl groups during PET under mild conditions, while simultaneously mitigating the formation of carboxylate by-products. However, most ionic liquid-catalyzed methanolysis of PET does not achieve complete conversion of PET into DMT; instead, it typically results in DMT yields of less than 95%. In addition, for most reported reactions, EG does not achieve continued upgrading during the reaction process. In this study, we optimized the ratio of DMC to methanol, exploiting the hydrogen bonding interactions between [EMIm][OAc] and the reactants to facilitate the methanolysis of PET. This adjustment not only facilitates the efficient depolymerization of PET under mild conditions but also enhances the ionic-liquid-catalyzed transesterification of EG with DMC. Consequently, the reaction equilibrium is shifted toward complete depolymerization with minimal methanol consumption, achieving a high DMT yield of 99%. In the development and technological advancement of the ionic liquid-assisted PET depolymerization strategy, this approach makes a certain contribution.

**2. Converting** **commercial mixed PET waste into DMT.**

| 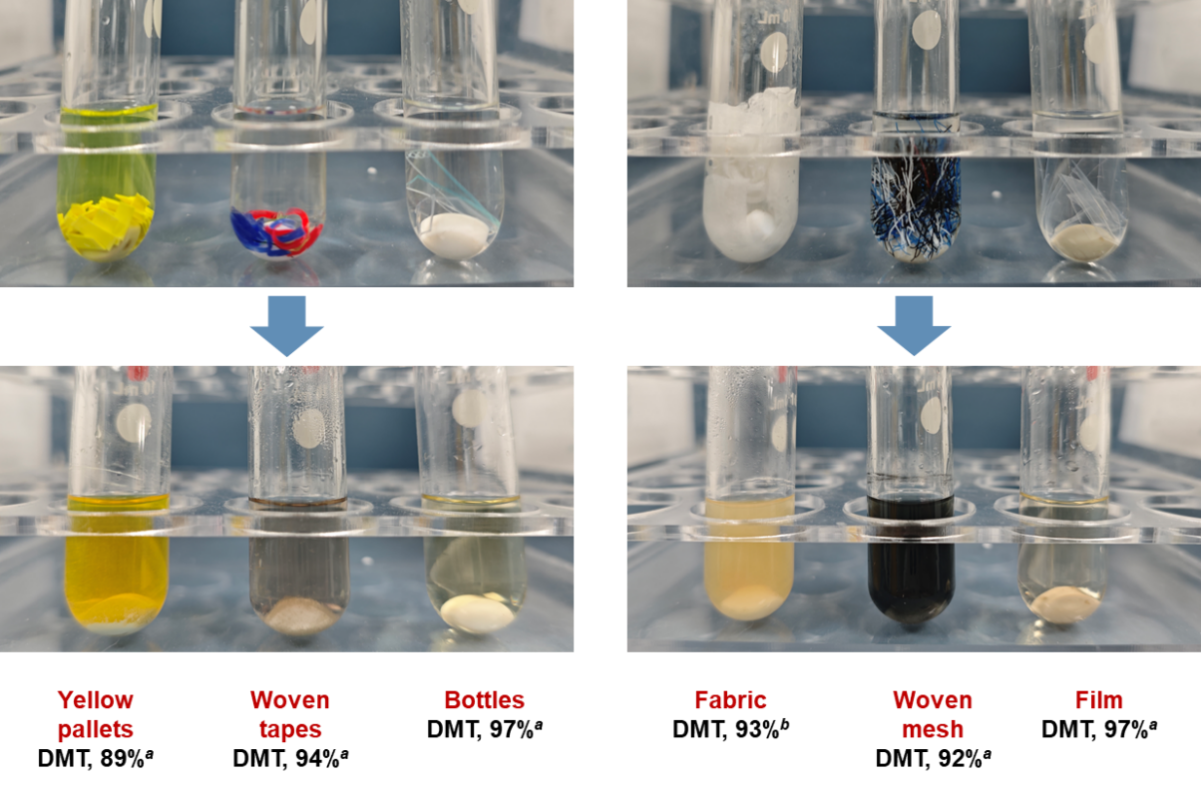 |
| --- |
| **Fig. S1.** *^a^* Standard reaction conditions: Waste PET (1 mmol), MeOH (w(CH_3_OH): w(polyester) = 1.3:1), [EMIm][OAc] (5 mol%), and DMC (3.5 mL) at 130°C for 3 h. Isolated yields. *^b^*at 130°C for 4 h. |

As illustrated in Fig. S1, the resulting reaction mixture from the woven tapes, bottles and transparent films was clear and free of precipitation or impurities. In contrast, the reaction mixtures from other commercial mixed PET material wastes (Yellow pallets, Fabric, and Woven mesh) exhibited some little impurities, which may be insoluble in various types of additives. Additionally, we performed a targeted separation of the product DMT by flash chromatography eluting hexanes/EtOAc. The isolated yield was shown in Fig. S1.

**3. The effect of water content on the catalytic system of PET depolymerization.**

| 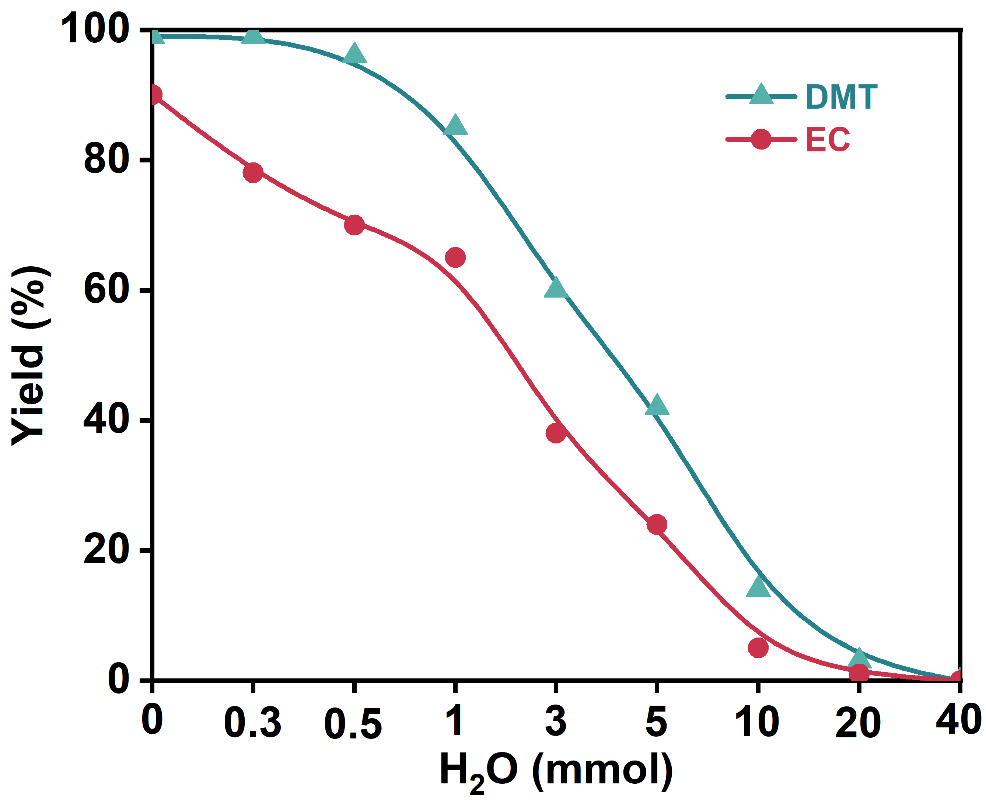 |
| --- |
| **Fig. S2.** *^a^* Standard reaction conditions: Waste PET (1 mmol), MeOH (w(CH_3_OH): w(polyester) = 1.3:1), [EMIm][OAc] (5 mol%), and DMC (3.5 mL) at 130°C for 2.5 h. |

As shown in Fig. S2, an increase in the amount of water gradually reduced the conversion of PET into both the DMT and EC, which was influenced by varying water contents. Specifically, the initial addition of water had a gradual negative impact on the yields of DMT and EC. Conversely, with further increases in the amount of water, the yields of all products experienced a rapid decline. Notably, when the amount of water reached 20 mmol, the depolymerization of PET was markedly insufficient. Trace amounts of both DMT and EC were detected upon the addition of 40 mmol of water. These results may be attributed to the fact that a small quantity of water has a minimal effect on the depolymerization of PET to yield DMT, while the presence of water may significantly enhance the hydrolysis of EC, leading to the production of methanol and EG. Furthermore, the presence of a large amount of water directly inhibits the progress of the PET methanolysis reaction.

**4. Characterization of the hydrogen bonds formed during the processes.**

**4.1.** **The structures of ILs used in this study.**

**Fig. S3.** The structures of ILs used in this study.

**4.2. ^1^H NMR characterizations of reactants.**

^
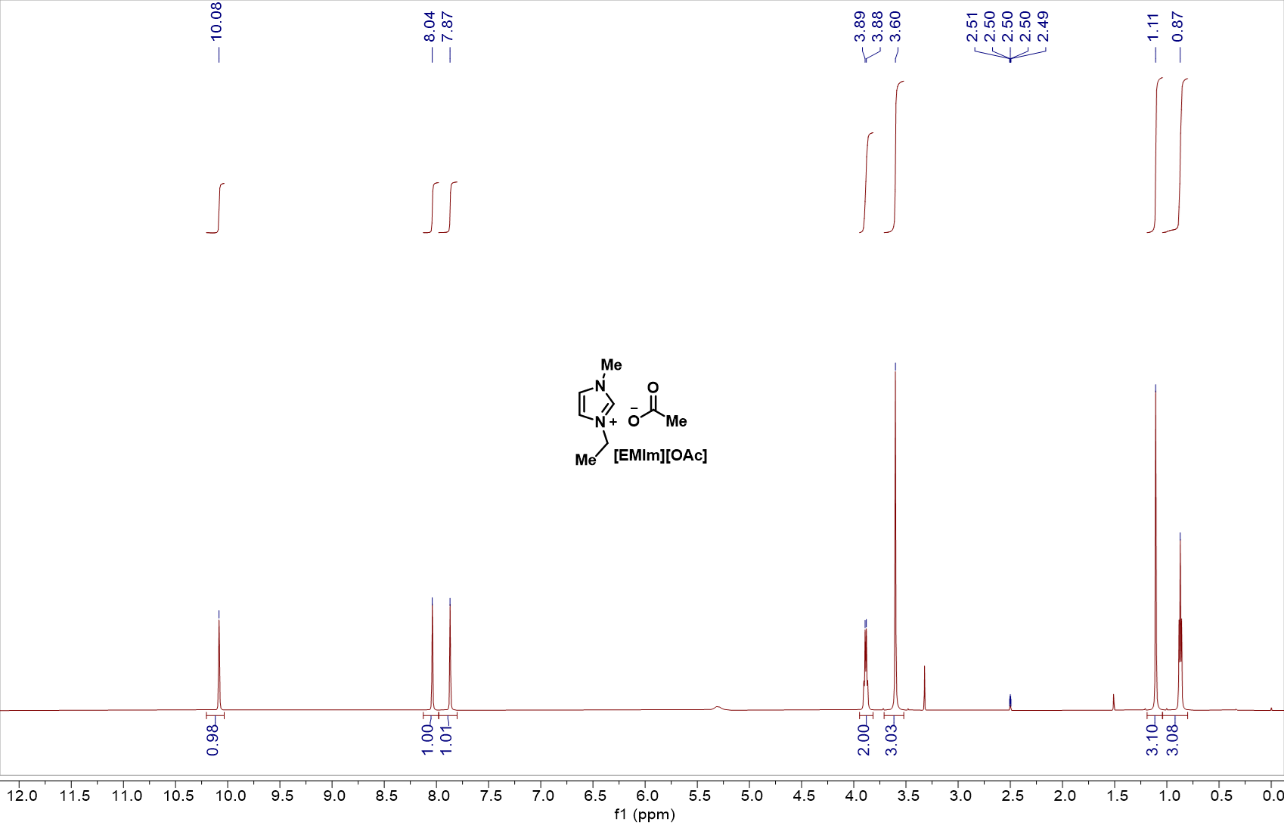
^

**Fig. S4.** ^1^H NMR spectra of [EMIm][OAc] in DMSO-*d_6_*.

^
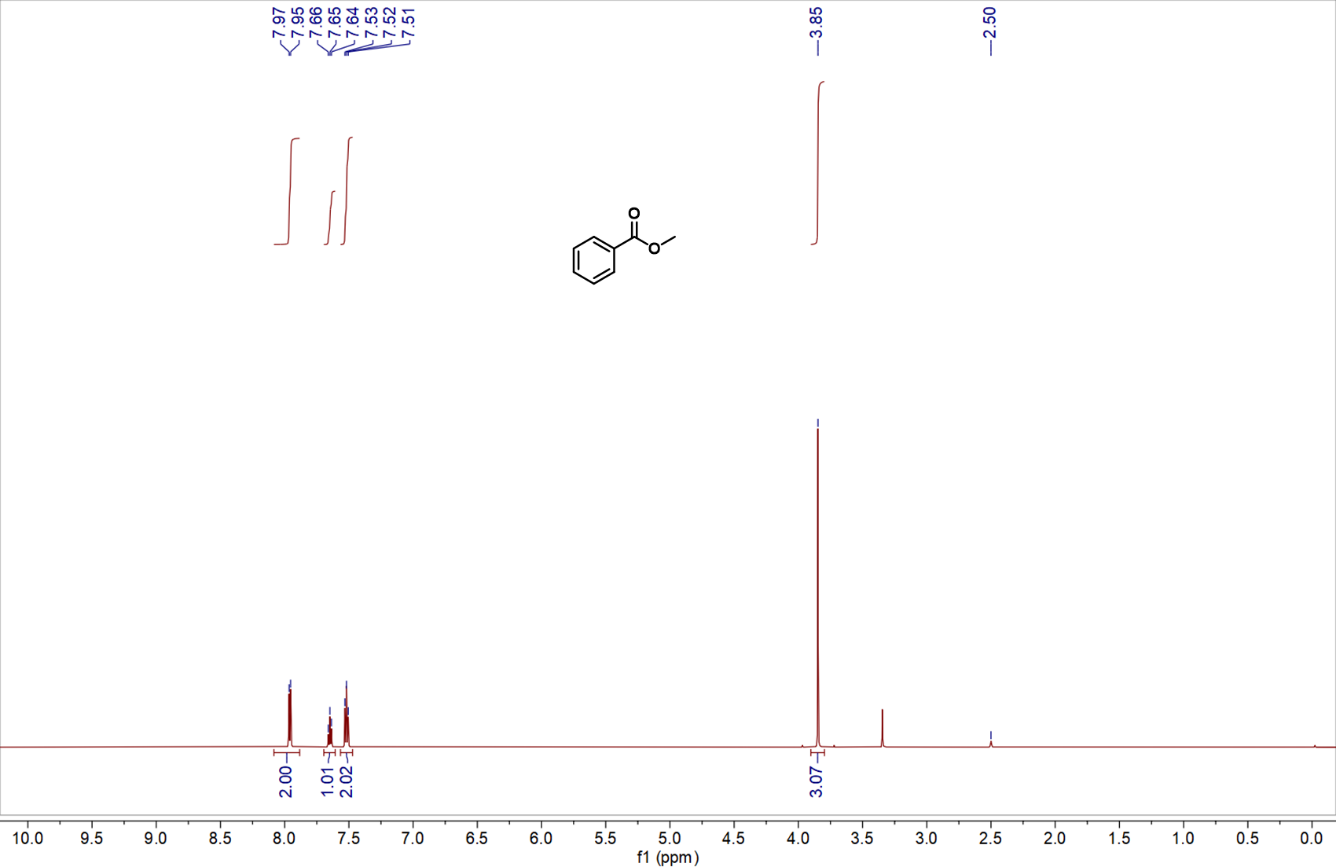
^

**Fig. S5.** ^1^H NMR spectra of methyl benzoate (MB) in DMSO-*d_6_*.

^
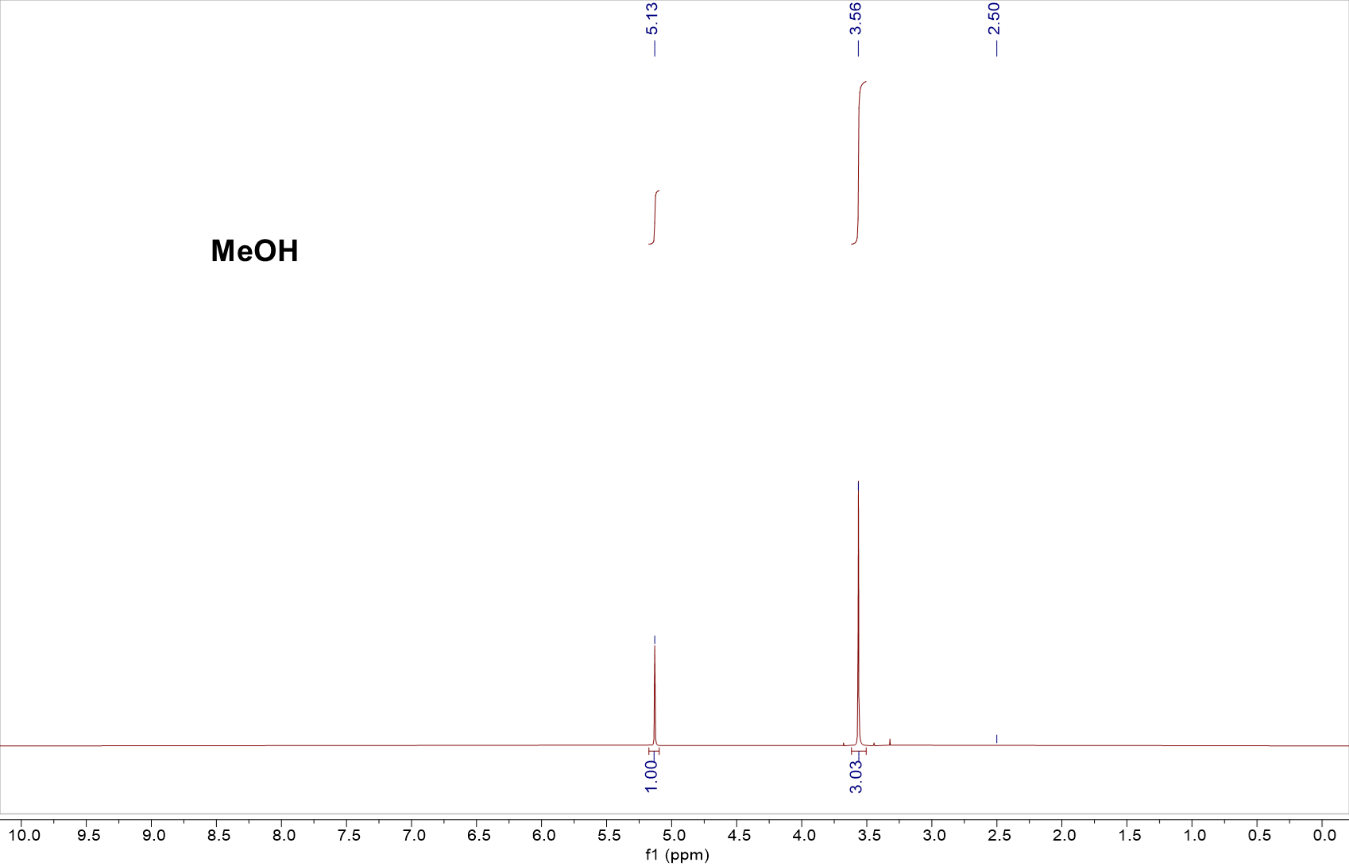
^

**Fig. S6.** ^1^H NMR spectra of MeOH in DMSO-*d_6_*.

^
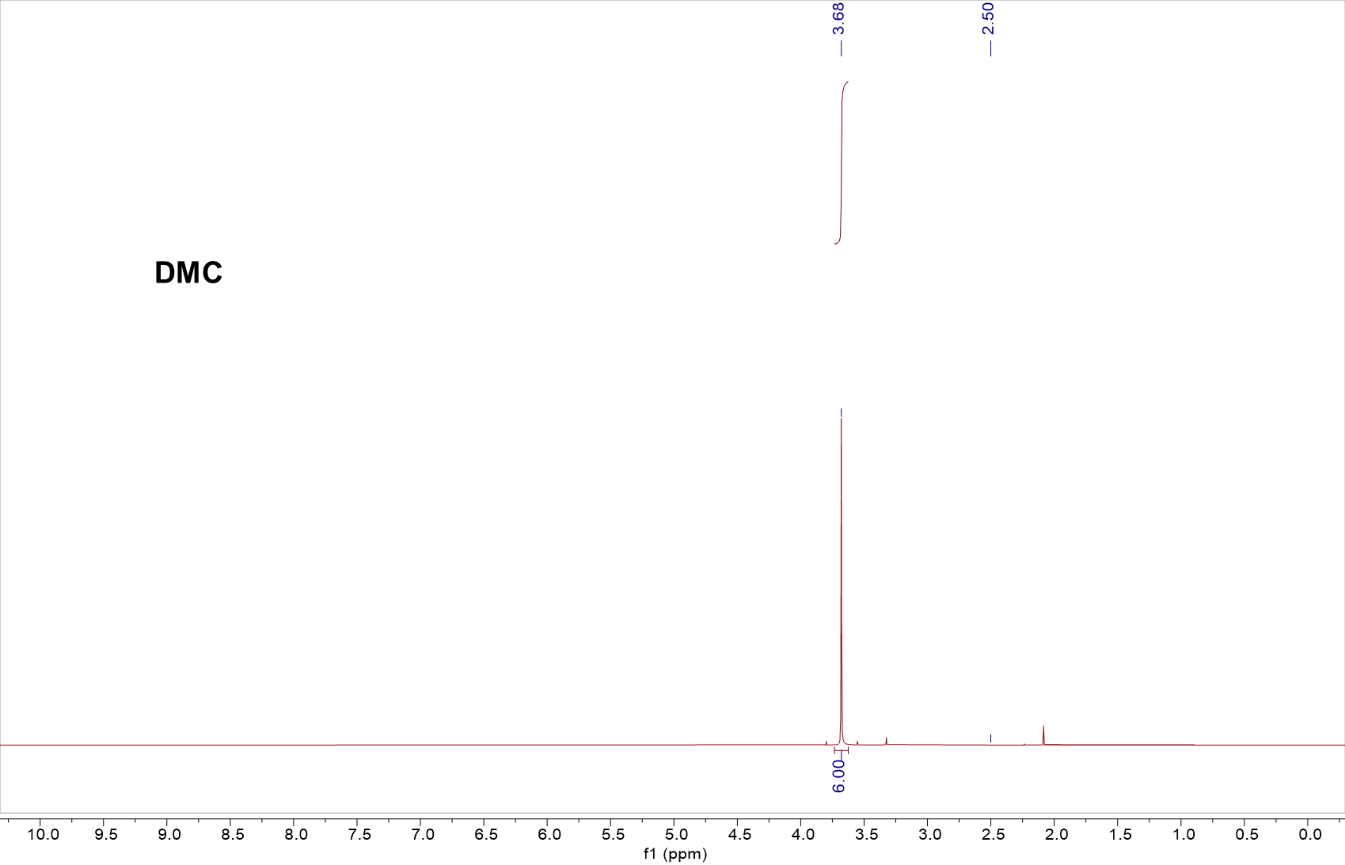
^

**Fig. S7.** ^1^H NMR spectra of dimethyl carbonate in DMSO-*d_6_*.

^
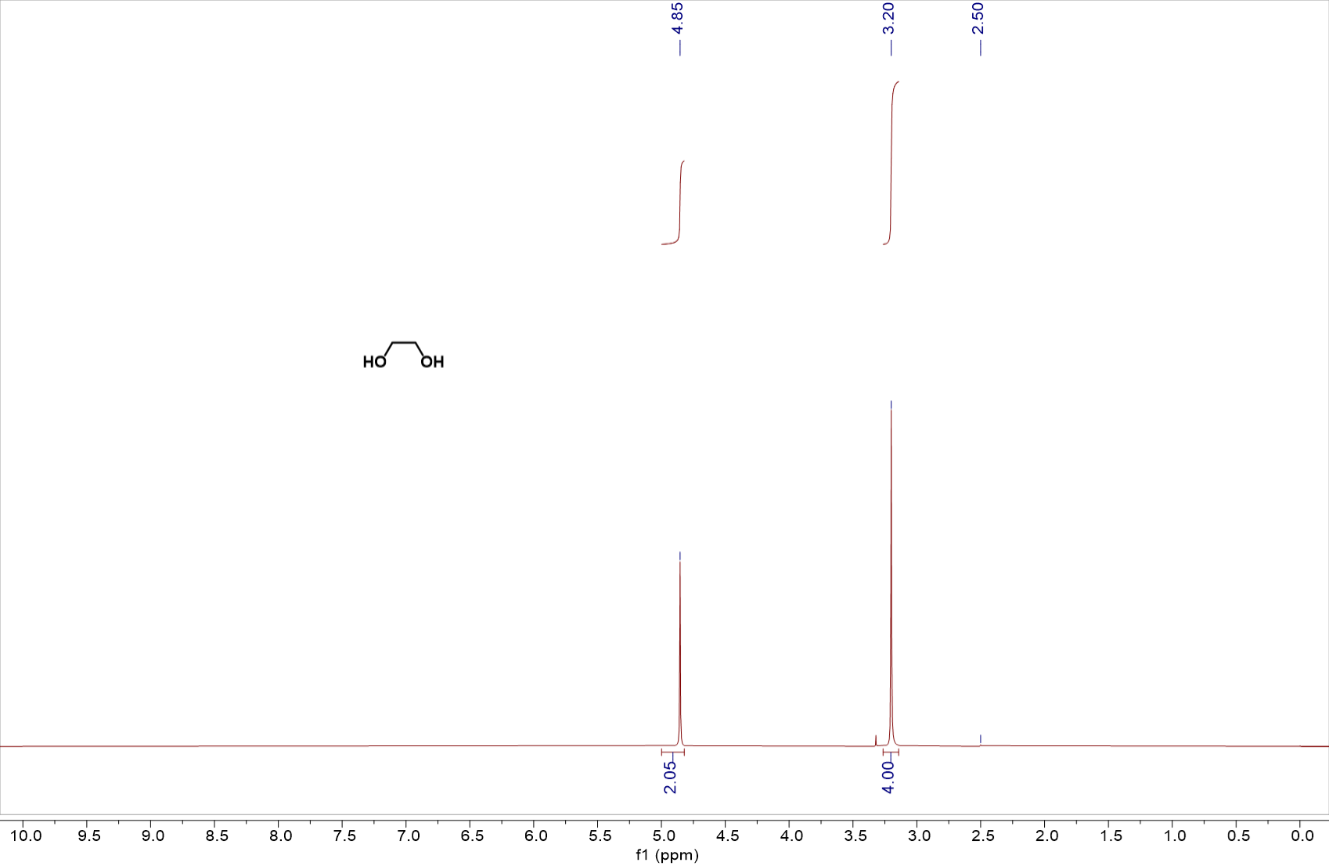
^

**Fig. S8.** ^1^H NMR spectra of ethylene glycol in DMSO-*d_6_*.

| 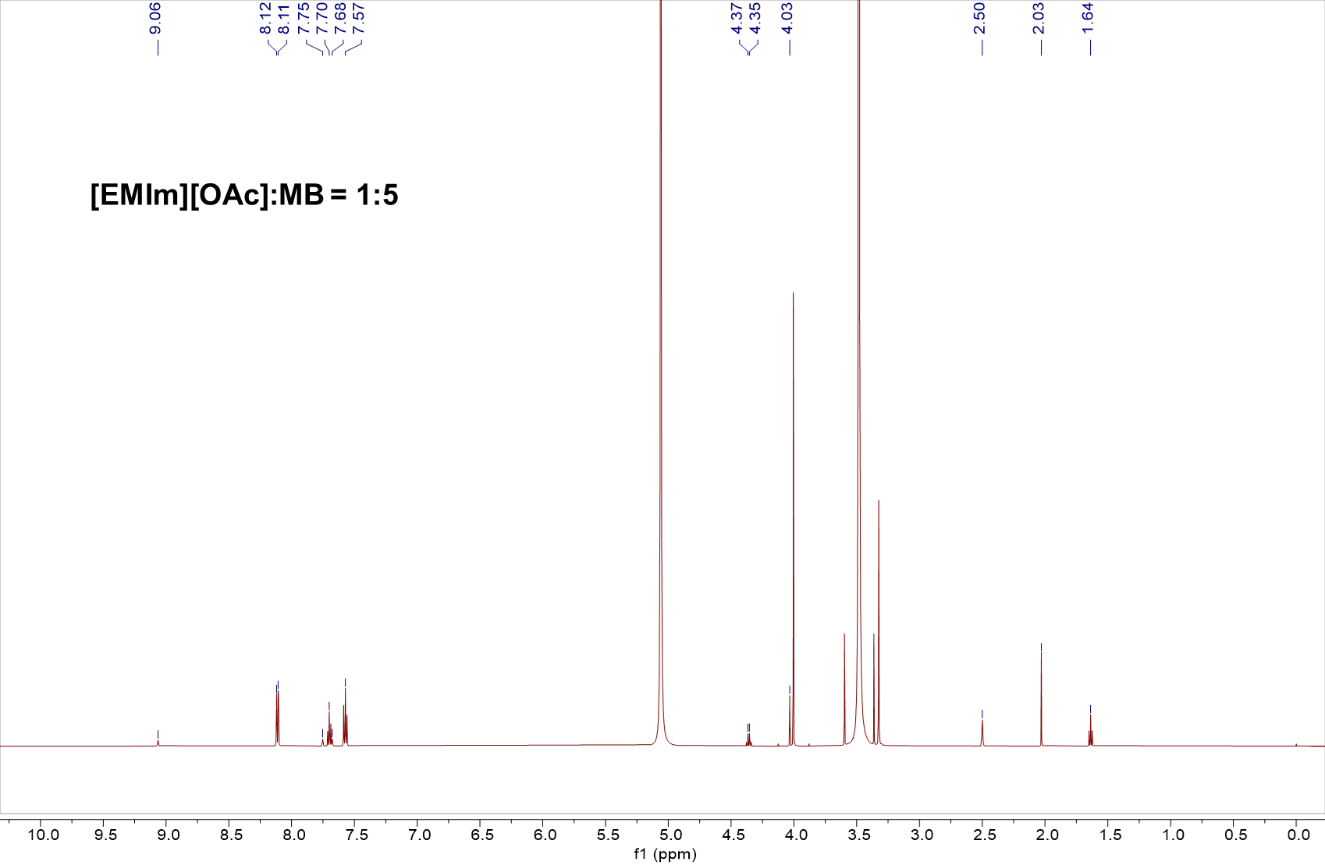   | | | | | | | | | |
| --- | --- | --- | --- | --- | --- | --- | --- | --- | --- |
| Different protons | C1-H | C2-H | C3-H | C4-H | C5-H | C6-H | C7-H | C8-10-H | C11-H |
|  | Chemical shift (ppm) | | | | | | | |  |
| Pure IL or MB | 4.11 | 9.14 | 4.45  4.44 | 1.72 | 7.76 | 7.83 | 2.08 | 8.12  7.70  7.57 | 4.09 |
| Mixture IL or MB | 4.03 | 9.06 | 4.37  4.35 | 1.50 | 7.68 | 7.75 | 2.03 | 8.20  7.79  7.66 | 4.03 |

**Fig. S9.** ^1^H NMR spectra of the mixture of MB and [EMIm][OAc] in DMSO-*d_6_*. Summary of H resonance signals of pure MB, [EMIm][OAc] and their mixture in methanol.

| 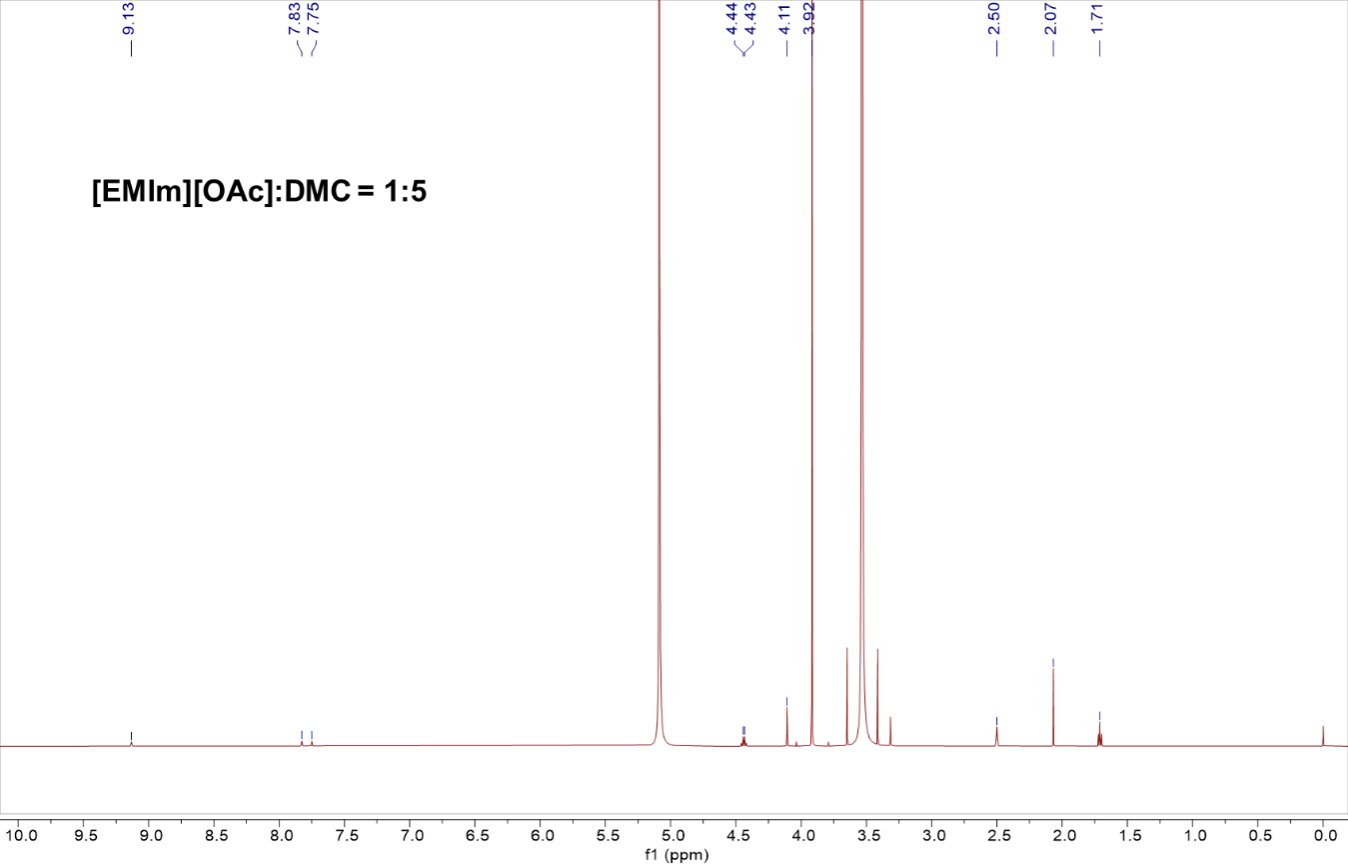   | | | | | | | | |
| --- | --- | --- | --- | --- | --- | --- | --- | --- |
| Different protons | C1-H | C2-H | C3-H | C4-H | C5-H | C6-H | C7-H | C8-H |
|  | Chemical shift (ppm) | | | | | | | |
| Pure IL or DMC | 4.11 | 9.14 | 4.45  4.44 | 1.72 | 7.76 | 7.83 | 2.08 | 3.94 |
| Mixture IL or DMC | 4.11 | 9.13 | 4.44  4.43 | 1.71 | 7.75 | 7.83 | 2.07 | 3.92 |

**Fig. S10.** ^1^H NMR spectra of the mixture of DMC and [EMIm][OAc] in DMSO-*d_6_*. Summary of H resonance signals of pure DMC, [EMIm][OAc] and their mixture in methanol.

| 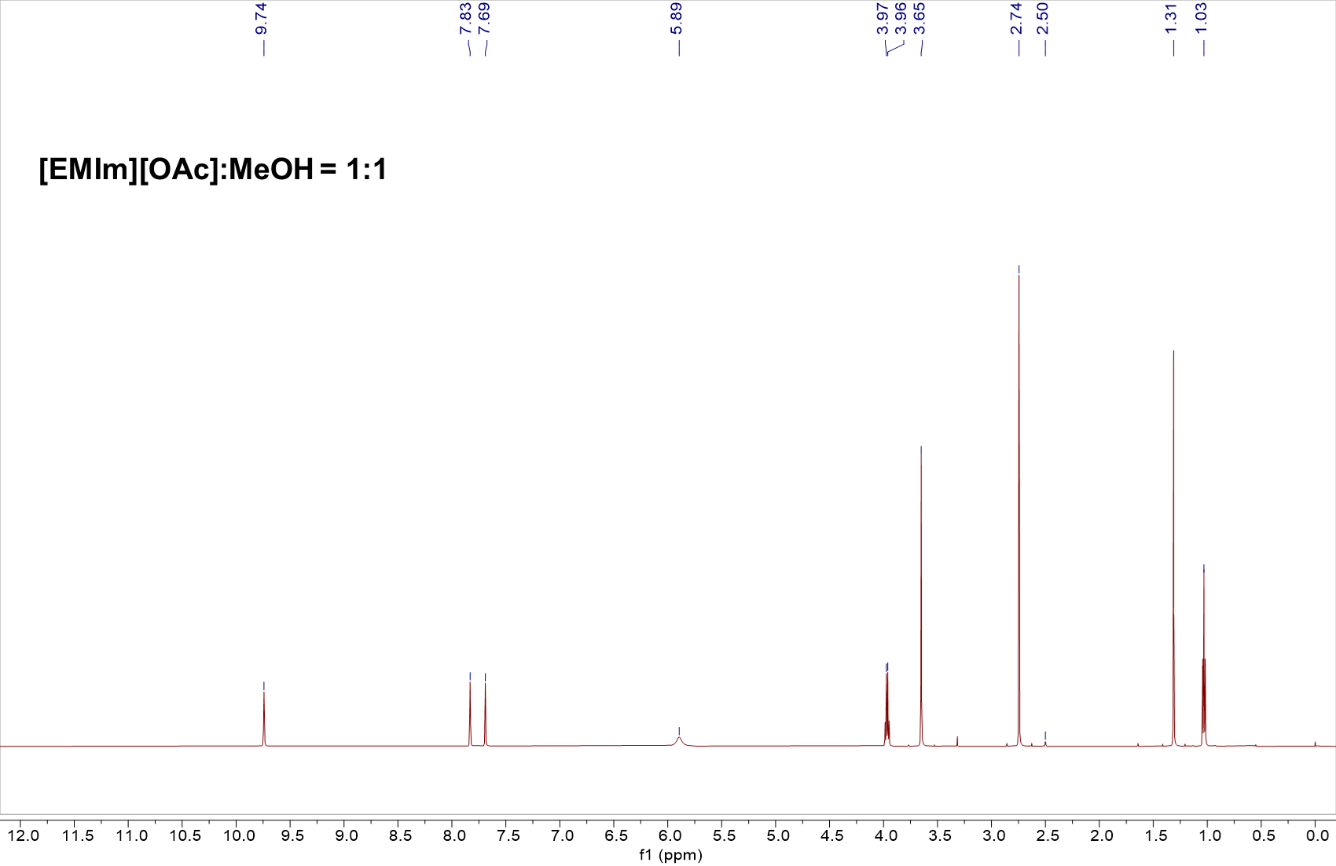   | | | | | | | | | |
| --- | --- | --- | --- | --- | --- | --- | --- | --- | --- |
| Different protons | C1-H | C2-H | C3-H | C4-H | C5-H | C6-H | C7-H | C8-H | C9-H |
|  | Chemical shift (ppm) | | | | | | | |  |
| Pure IL or MeOH | 3.60 | 10.08 | 3.89  3.88 | 0.87 | 7.87 | 8.04 | 1.11 | 3.56 | 5.13 |
| Mixture IL or MeOH | 3.65 | 9.74 | 3.96  3.97 | 1.03 | 7.69 | 7.83 | 1.31 | 2.74 | 5.89 |

**Fig. S11.** ^1^H NMR spectra of the mixture of MeOH and [EMIm][OAc] in DMSO-*d_6_*. Summary of H resonance signals of pure MeOH, [EMIm][OAc] and their mixture.

| 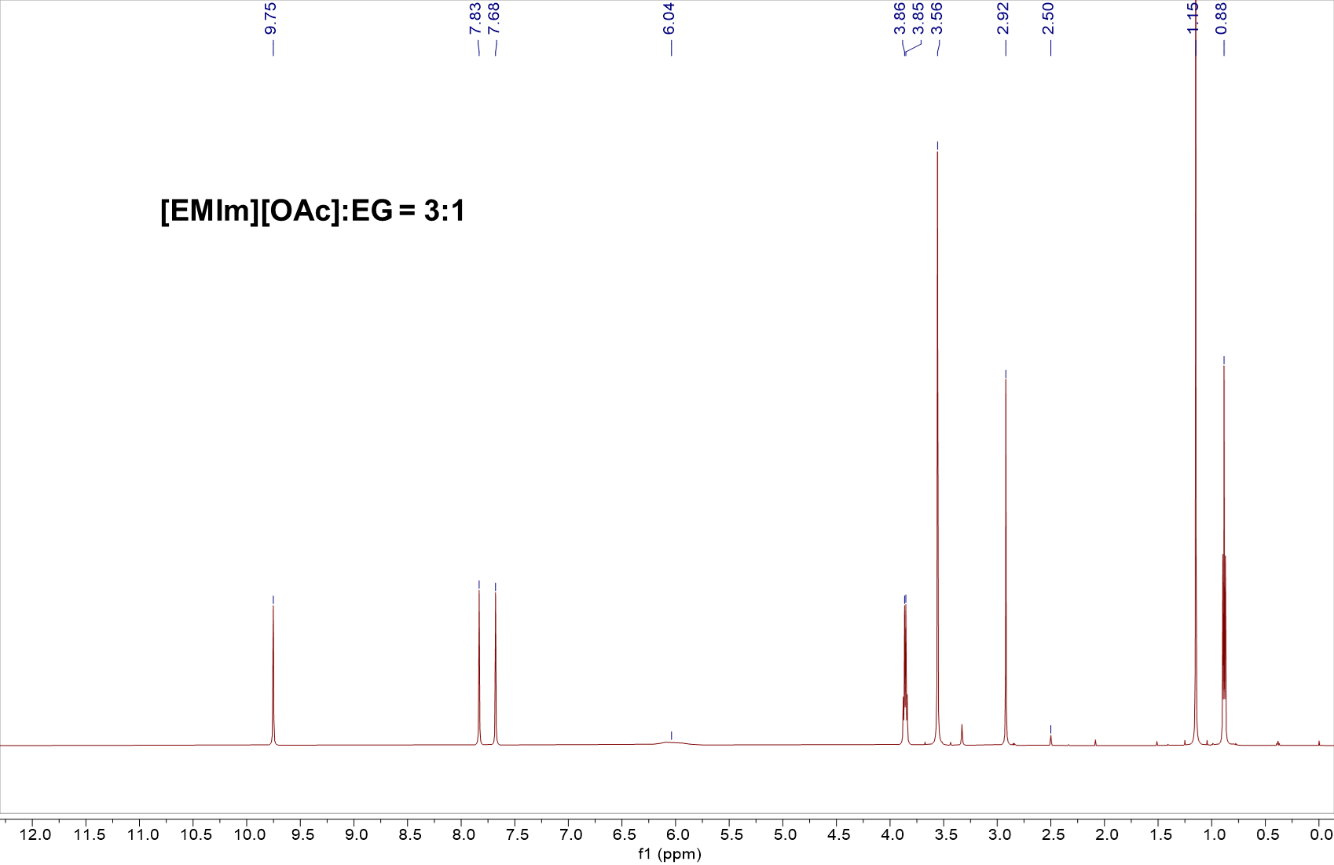   | | | | | | | | | |
| --- | --- | --- | --- | --- | --- | --- | --- | --- | --- |
| Different protons | C1-H | C2-H | C3-H | C4-H | C5-H | C6-H | C7-H | C8-H | C9-H |
|  | Chemical shift (ppm) | | | | | | | |  |
| Pure IL or EG | 3.60 | 10.08 | 3.89  3.88 | 0.87 | 7.87 | 8.04 | 1.11 | 3.2 | 4.85 |
| Mixture IL or EG | 3.56 | 9.75 | 3.86  3.85 | 0.88 | 7.68 | 7.83 | 1.15 | 2.92 | 6.04 |

**Fig. S12.** ^1^H NMR spectra of the mixture of EG and [EMIm][OAc] in DMSO-*d_6_*. Summary of H resonance signals of pure EG, [EMIm][OAc] and their mixture.

**4.3. ^1^H NMR characterization of obtained DMT.**


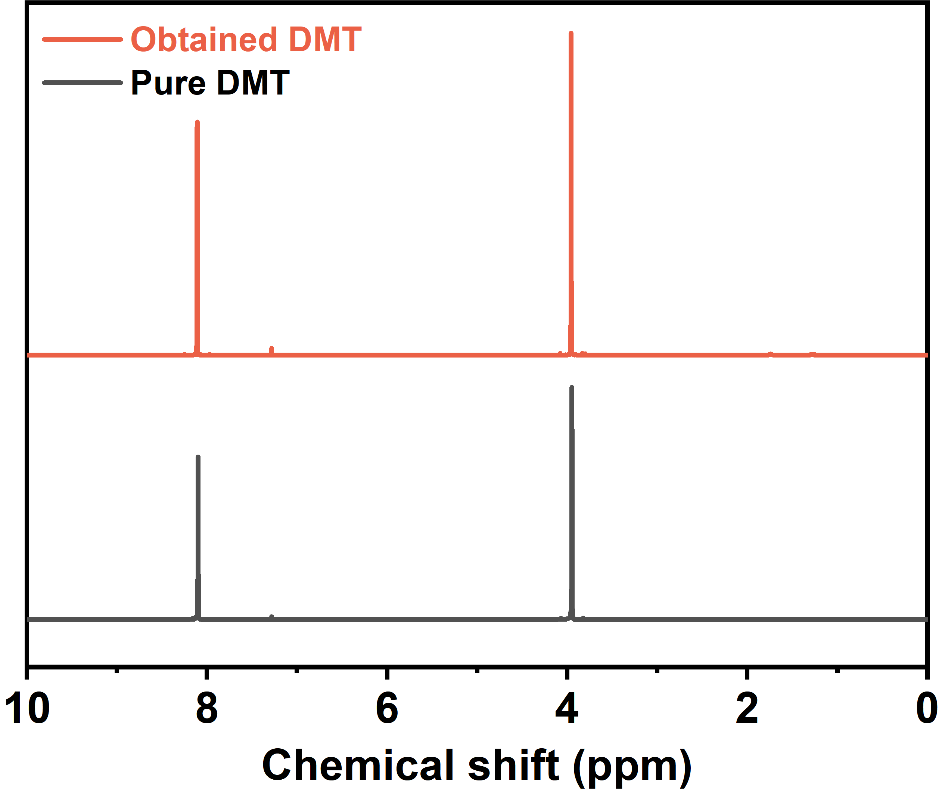


**Fig. S13.** ^1^H NMR patterns of the obtained DMT and the pure DMT.

**4.4. ^1^H NMR characterization of products.**


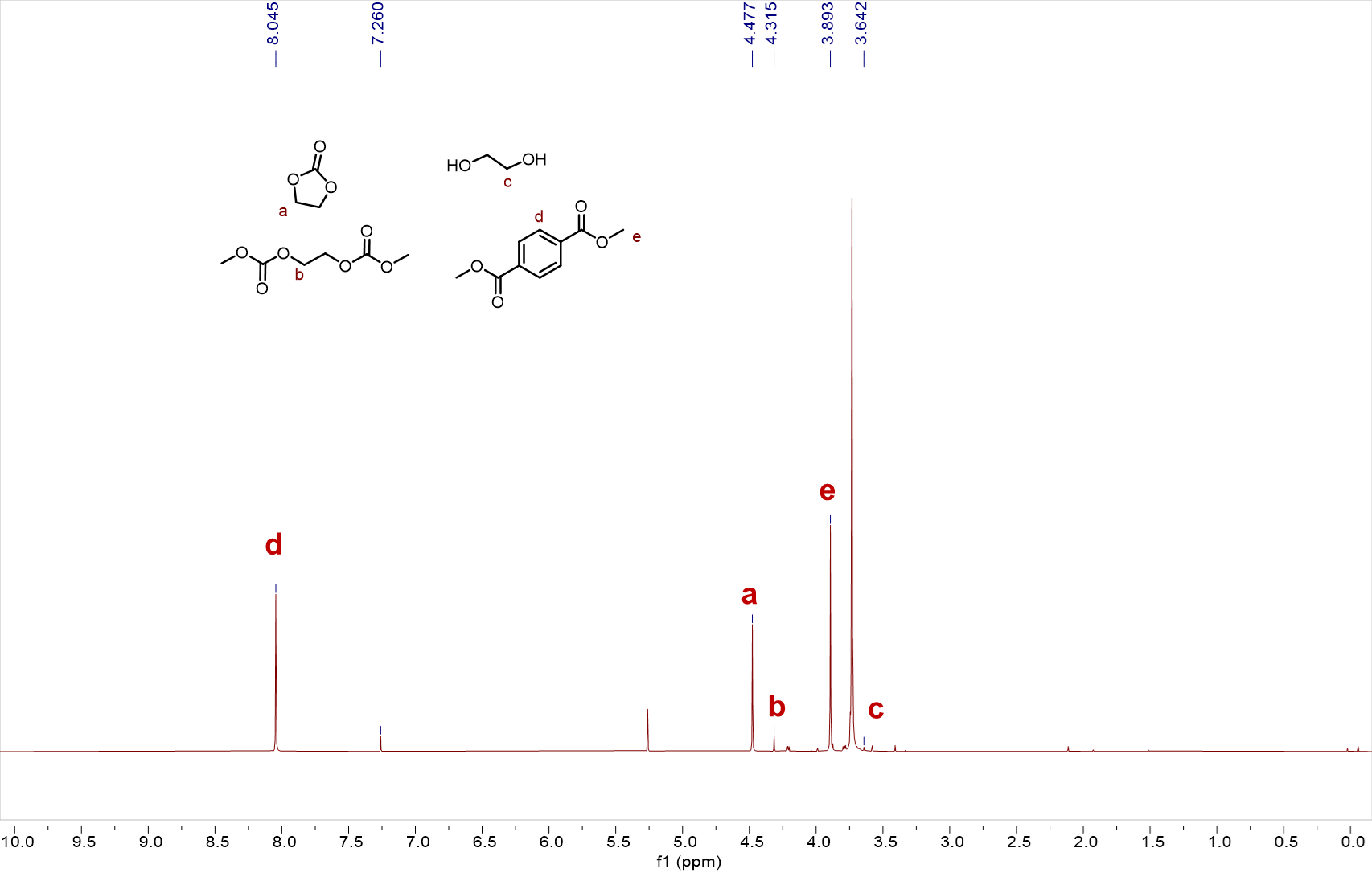


**Fig. S14.** ^1^H NMR patterns of the reaction mixture.


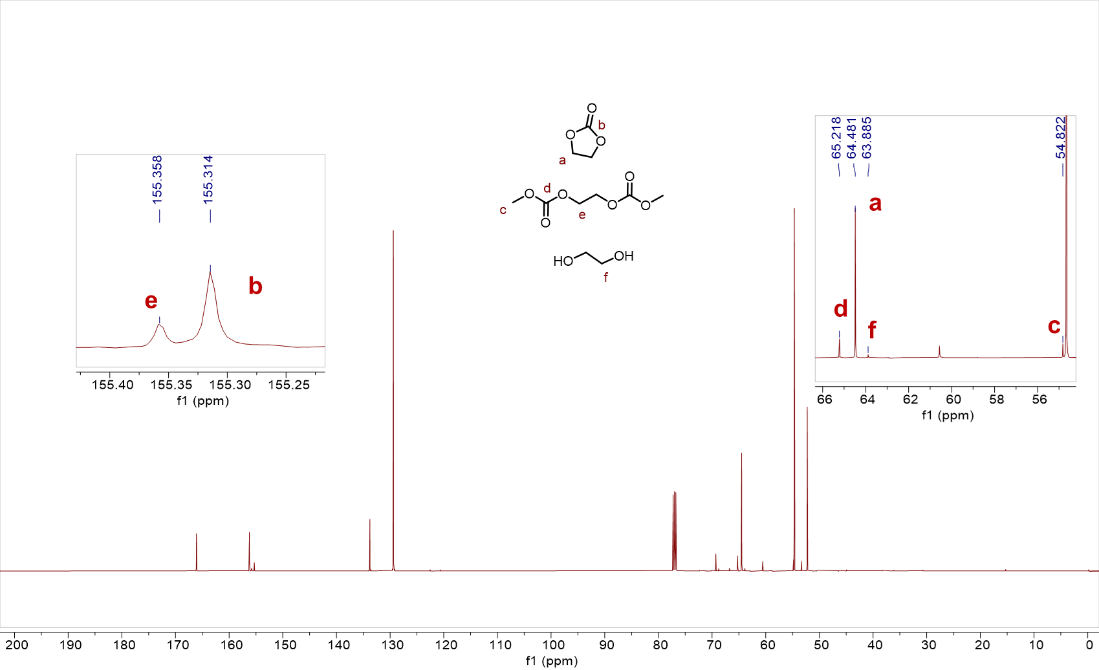


**Fig. S15.** ^13^C NMR patterns of the reaction mixture.

**5. ^1^H NMR and ^13^C NMR data of all compounds.**

Dimethyl terephthalate (DMT) [16]: white solid, GC yield 99%; ^1^H NMR (600 MHz, CDCl_3_) δ = 8.08 (s, 4H), 3.92 (s, 6H); ^13^C NMR (125 MHz, CDCl_3_) δ = 166.3, 133.9, 129.5, 65.9, 52.4.

**6. ^1^H NMR and ^13^C NMR spectra of all compounds.**

**Fig. S16.** ^1^H NMR of DMT (600 MHz, CDCl_3_) and ^13^C NMR of DMT (151 MHz, CDCl_3_).

**References**

1. H. Kurokawa, M. Ohshima, K. Sugiyama, H. Miura, Methanolysis of polyethylene terephthalate (PET) in the presence of aluminium tiisopropoxide catalyst to form dimethyl terephthalate and ethylene glycol, Polym. Degrad. Stabil. 79 (2003) 529-533, <https://doi.org/10.1016/s0141-3910(02)00370-1>.
2. S. Mishra, A.S. Goje, Kinetic and thermodynamic study of methanolysis of poly(ethylene terephthalate) waste powder, Polym Int. 52 (2003) 337-342, <https://doi.org/10.1002/pi.1147>.
3. J.-T. Du, Q. Sun, X.-F. Zeng, D. Wang, J.-X. Wang, J.-F. Chen, ZnO nanodispersion as pseudohomogeneous catalyst for alcoholysis of polyethylene terephthalate, Chem. Eng. Sci. 220 (2020) 115642, <https://doi.org/10.1016/j.ces.2020.115642>.
4. P. Duong Dinh, J. Cho, Low-energy catalytic methanolysis of poly(ethyleneterephthalate), Green Chem. 23 (2021) 511-525, <https://doi.org/10.1039/d0gc03536j>.
5. Z.T. Laldinpuii, C. Lalmuanpuia, S. Lalhmangaihzuala, V. Khiangte, Z. Pachuau, K. Vanlaldinpuia, Biomass waste-derived recyclable heterogeneous catalyst for aqueous aldol reaction and depolymerization of PET waste, New J. Chem. 45 (2021) 19542-19552, <https://doi.org/10.1039/d1nj03225a>.
6. S. Tanaka, J. Sato, Y. Nakajima, Capturing ethylene glycol with dimethyl carbonate towards depolymerisation of polyethylene terephthalate at ambient temperature, Green Chem. 23 (2021) 9412-9416, <https://doi.org/10.1039/d1gc02298a>.
7. Z.T. Laldinpuii, V. Khiangte, S. Lalhmangaihzuala, C. Lalmuanpuia, Z. Pachuau, C. Lalhriatpuia, et al., Methanolysis of PET waste using heterogeneous catalyst of bio-waste origin, J. Polym. Environ. 30 (2022) 1600-1614, <https://doi.org/10.1007/s10924-021-02305-0>.
8. B. Ye, R. Zhou, Z. Zhong, S. Wang, H. Wang, Z. Hou, Upcycling of waste polyethylene terephthalate to dimethyl terephthalate over solid acids under mild conditions, Green Chem. 25 (2023) 7243-7252, <https://doi.org/10.1039/d3gc02051g>.
9. S. Tang, F. Li, J. Liu, B. Guo, Z. Tian, J. Lv, Calcined sodium silicate as solid base catalyst for alcoholysis of poly(ethylene terephthalate), J. Chem. Technol. Biotechnol. 97 (2022) 1305-1314, <https://doi.org/10.1002/jctb.7025>.
10. S. Tang, F. Li, J. Liu, B. Guo, Z. Tian, J. Lv, MgO/NaY as modified mesoporous catalyst for methanolysis of polyethylene terephthalate wastes, J. Environ. Chem. Eng. 10 (2022) 107927, <https://doi.org/10.1016/j.jece.2022.107927>.
11. J. Tang, X. Meng, X. Cheng, Q. Zhu, D. Yan, Y. Zhang, et al., Mechanistic insights of cosolvent efficient enhancement of PET methanol alcohololysis, Ind. Eng. Chem. Res. 62 (2023) 4917-4927, <https://doi.org/10.1021/acs.iecr.2c04419>.
12. M. Liu, J. Guo, Y. Gu, J. Gao, F. Liu, Versatile imidazole-anion-derived ionic liquids with unparalleled activity for alcoholysis of polyester wastes under mild and green conditions, ACS Sustain. Chem. Eng. 6 (2018) 15127-15134, <https://doi.org/10.1021/acssuschemeng.8b03591>.
13. Z. Jiang, D. Yan, J. Xin, F. Li, M. Guo, Q. Zhou, et al., Poly(ionic liquid)s as efficient and recyclable catalysts for methanolysis of PET, Polym. Degrad. Stabil. 199 (2022) 109905, <https://doi.org/10.1016/j.polymdegradstab.2022.109905>.
14. M. Ma, S. Wang, Y. Liu, H. Yu, S. Yu, C. Ji, et al., Insights into the depolymerization of polyethylene terephthalate in methanol, J. Appl. Polym. Sci. 139 (2022) e52814, <https://doi.org/10.1002/app.52814>.
15. X.L. Qu, G.Y. Zhou, R. Wang, B.L. Yuan, M. Jiang, J. Tang, Synergistic catalysis of imidazole acetate ionic liquids for the methanolysis of spiral poly(ethylene 2,5-furandicarboxylate) under a mild condition, Green Chem. 23 (2021) 1871-1882, <https://doi.org/10.1039/d0gc04019c>.
16. Q. Jiang, A. Zhao, B. Xu, J. Jia, X. Liu, C.C. Guo, PIFA-mediated esterification reaction of alkynes with alcohols via oxidative cleavage of carbon triple bonds, J. Org. Chem. 79 (2014) 2709-2715, <https://doi.org/10.1021/jo5003517>.
